# Response-dependent dynamics of cell-specific inhibition in cortical networks *in vivo*

**DOI:** 10.1038/ncomms6689

**Published:** 2014-12-11

**Authors:** Sami El-Boustani, Mriganka Sur

**Affiliations:** 1 Department of Brain and Cognitive Sciences, Picower Institute for Learning and Memory, Massachusetts Institute of Technology, 77 Massachusetts Avenue, Cambridge, Massachusetts 02139, USA

## Abstract

In the visual cortex, inhibitory neurons alter the computations performed by target cells via combination of two fundamental operations, division and subtraction. The origins of these operations have been variously ascribed to differences in neuron classes, synapse location or receptor conductances. Here, by utilizing specific visual stimuli and single optogenetic probe pulses, we show that the function of parvalbumin-expressing and somatostatin-expressing neurons in mice *in vivo* is governed by the overlap of response timing between these neurons and their targets. In particular, somatostatin-expressing neurons respond at longer latencies to small visual stimuli compared with their target neurons and provide subtractive inhibition. With large visual stimuli, however, they respond at short latencies coincident with their target cells and switch to provide divisive inhibition. These results indicate that inhibition mediated by these neurons is a dynamic property of cortical circuits rather than an immutable property of neuronal classes.

## Abstract (web summary)

Inhibitory neurons in the visual cortex alter the computations of target cells by exerting division or subtraction effects, but what determines these different functions is not clear. Here the authors use visual stimuli and optogenetics to show that the effects mediated by somatostatin-expressing and parvalbumin-expressing neurons are driven by their response mode and timing.

## Graphical abstract

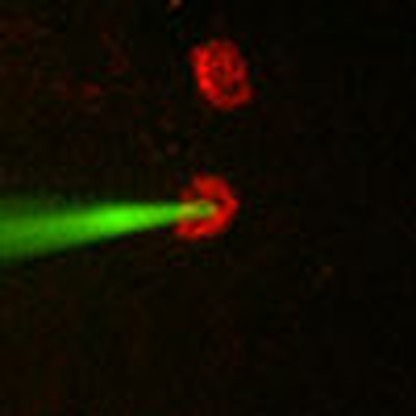

Arithmetic operations such as division and subtraction are a fundamental and widespread property of inhibition in neuronal networks^1^,^2^,^3^. Divisive inhibition, a form of gain control, plays a major part in scaling the response amplitude of neurons while keeping their sensory selectivity or function intact^4^,^5^. Response scaling has been shown to occur during diverse functions such as directed visual attention^6^,^7^,^8^, contrast-invariant orientation selectivity^9^,^10^, multisensory integration^11^ and value estimation^12^. In contrast, subtractive inhibition can sharpen the selectivity of neurons^13^,^14^, possibly increasing the discrimination capability of cell populations and thereby behavioural performance^15^.

It has been suggested that different cortical inhibitory cell classes provide distinct combinations of divisive or subtractive inhibition during stimulus-mediated synaptic drive *in vivo*^16^,^17^,^18^,^19^. Although representing a minority of cortical cells, GABAergic neurons are highly diverse in their molecular, morphological and functional properties^20^,^21^, and can therefore contribute in various ways to the computational capability of cortical circuits^22^,^23^,^24^. Recent studies in visual cortex using optogenetic activation of inhibitory neurons have provided important insights into their function. In particular, parvalbumin-expressing (PV+) and somatostatin-expressing (SOM+) inhibitory neurons—the two key neuron classes that inhibit pyramidal neurons^25^—have been shown to alter responses of layer 2/3 cortical pyramidal neurons with distinct suppressive patterns displaying either divisive or subtractive inhibition^14^,^15^,^26^. The specific role of these inhibitory neuron classes, however, remains unclear: two studies found that, in visual cortex, PV+ neurons can alter the response gain of their targets^14^,^26^ but with little effect on orientation tuning width, whereas one study found that PV+ neuron activation could alter tuning width significantly^15^. Similarly, SOM+ neurons have been shown to have either a subtractive effect^14^ or a divisive effect^15^, leading in the former case to an increase in orientation selectivity and reduction in tuning width and in the latter case to no change in tuning width. Differences regarding the role of PV+ and SOM+ neurons could be imputed to differences in optogenetic stimulation protocols^27^, suggesting that the functions performed by different inhibitory neurons is not a fixed property of cortical networks but is a consequence of more complex dynamics^28^.

The principles that govern the dynamics of PV+ and SOM+ neurons as well as the mechanisms underlying their function are still unknown. One proposal is that PV+ neurons, which target the soma and proximal dendrites of pyramidal neurons, recruit different conductances compared with SOM+ neurons, which mainly target distal dendrites^21^,^29^,^30^. However, to our knowledge, no experimental or theoretical evidence exists demonstrating that these structural or biophysical properties are essential for specific and diverse functional operations of inhibitory neurons. An alternative proposal is that inhibition can dynamically influence excitation depending on the temporal relationship between the two^31^,^32^; roles of inhibitory neurons can therefore be altered as a function of their firing coordination with target neurons.

When driven with specific stimulus ensembles, inhibitory neurons can display distinct response modes, as defined by their latency, amplitude and selectivity^33^,^34^. Here we have examined the hypothesis that different temporal response modes of inhibitory neurons dynamically shape their function. Our study design differed from previous studies of cell-specific function in two important respects. First, to probe the nature and timing of responses, we employed briefly flashed visual stimuli and contrasting stimulus ensembles that evoked different response dynamics in inhibitory neurons. Second, to minimize the additive effects of optogenetic stimulation on visual responses, we employed precisely timed single optogenetic pulses to interrogate the nature of inhibition. By recording from identified target neurons in mouse primary visual cortex (V1), we show that the response timing and profile of inhibitory neurons, and in particular SOM+ neurons, indeed shapes the operations they perform. A computational model shows that such response modes arise from simple rules of network connectivity and synaptic summation, yet enable dynamic switching of function between subtraction and division. These findings help to explain many of the diverse results described previously for inhibitory neuron function.

## Results

### V1 neuron responses to sparse noise and full-field stimuli

To examine the role of specific inhibitory neuron classes in V1 as a function of stimulus properties and response features, we used two different types of visual stimuli. In a sparse noise protocol, which is conventionally used to map neuronal receptive fields^33^,^35^ (see Methods), the location of the stimulus (a square patch of light) and its polarity (ON or OFF) changed from one frame to the next. In a full-field protocol, stimulus flashes (covering the entire visual field) were displayed at different intensities. These stimuli were thus spatially uniform but had variable contrasts compared with offset periods (Supplementary Fig. 1). Sparse noise and full-field stimuli provide complementary ways to rapidly probe the spatial and temporal summation properties of neurons and their receptive fields.

**Figure 1:**
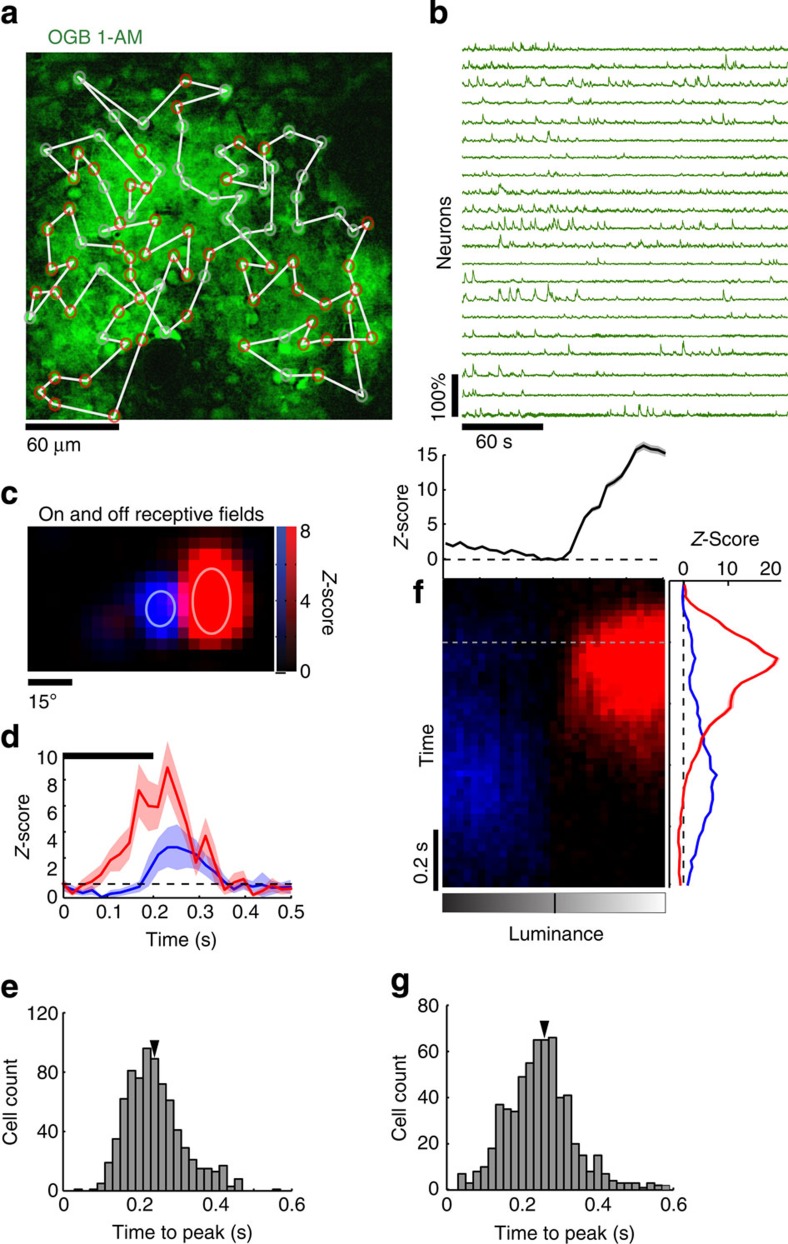
Neuronal spikes, receptive fields and intensity response curves as revealed by calcium imaging. (**a**) Baseline image used to identify the location of cells loaded with OGB-1AM. The two-photon laser scan-path is overlaid in white on top of the image. Cells displaying significant visual responses are marked with red circles. (**b**) Examples of calcium traces of neurons that were imaged through the scan-path. (**c**) Spatial ON (red) and OFF (blue) receptive fields of an example neuron obtained with calcium imaging in response to sparse noise stimuli. Gaussian fits are represented by ellipses in light colours. (**d**) Averaged evoked responses to the complete sparse noise sequence for ON (red) and OFF (blue) stimuli. Dashed line indicates the baseline spontaneous activity and the bold black line indicates the duration of stimulus presentation. S.e.m. values are shown as shaded areas. (**e**) Response time-to-peak distribution for all neurons displaying a significant sensory response (*n*=761, the arrow indicating the average: 0.24 s). (**f**) Neuron population responses to full-field flashes of various luminance intensities as a function of time. The curves on the right margin indicate the average response time course for ON (red) or OFF (blue) stimuli. The intensity curve on top is the population *Z*-score estimated around the dashed grey line (200 ms±100 ms). S.e.m. values are shown as shaded areas. (**g**) Population distributions over all recorded neurons with significant responses to full-field flashes showing response time-to-peak (*n*=620, average at 0.27 s).

We first characterized the responses of V1 neurons to these stimuli using 2-photon calcium imaging with the calcium indicator OGB-1AM. Using a fast-scan strategy, we were able to image numerous neurons sequentially at high acquisition rate^14^ (Fig. 1a,b, see Methods). To evaluate the spatio-temporal properties of these cells, we estimated their instantaneous firing rate from the calcium traces using a fast deconvolution algorithm^36^. The validity of the estimated firing rate was illustrated through targeted cell-attached recordings of an OGB-filled neuron during simultaneous calcium imaging (Supplementary Fig. 2). To facilitate the comparison between electrophysiological recordings and calcium imaging, we used normalized unit-less statistics and displayed response magnitudes as *Z*-scores (see Methods).

In response to sparse noise, neurons displayed a classical receptive field with ON and OFF subfields corresponding to their responses to bright or dark squares, respectively (Fig. 1c). Most of the recorded cells displayed a strong ON field with either no OFF field or a weaker and delayed OFF response (Fig. 1c,d; Supplementary Fig. 3). Similar asymmetrical representation of ON and OFF sensitivity has been previously observed in mouse V1 (ref. 35) and in adult macaque V1 superficial layers^37^. We therefore based most of our analysis on ON responses. Among all the imaged neurons, 39% (761 out of 1,928 neurons, *Z*-score >3, 16 animals) responded significantly to sparse noise, with latencies to peak centred around 0.24 s (Fig. 1e). Experiments where a large fraction of cells displayed strong calcium signals revealed details of the retinotopic mapping and functional organization of mouse V1, in line with a previous report where a different stimulus ensemble was used to map receptive fields in mouse V1 (ref. 38) (Supplementary Fig. 4). When full-field flashes were presented, neurons responded in a monotonically increasing way to ON and OFF stimuli. OFF responses were on average weaker and delayed, similar to the responses with sparse noise (Fig. 1f). For this stimulus ensemble, 41% of all neurons (620 out of 1,518 neurons, *Z*-score >3, 8 animals) responded significantly, and comparable to sparse noise stimulation, latencies to peak were centred around 0.27 s (Fig. 1g). Thus, sparse noise and full-field stimuli provide robust characterization of neuronal receptive fields, along with measures of response magnitude and timing as probes of potential temporal interactions between inhibitory neurons and their targets.

### Distinct functional responses of PV+ and SOM+ neurons

**Figure 2:**
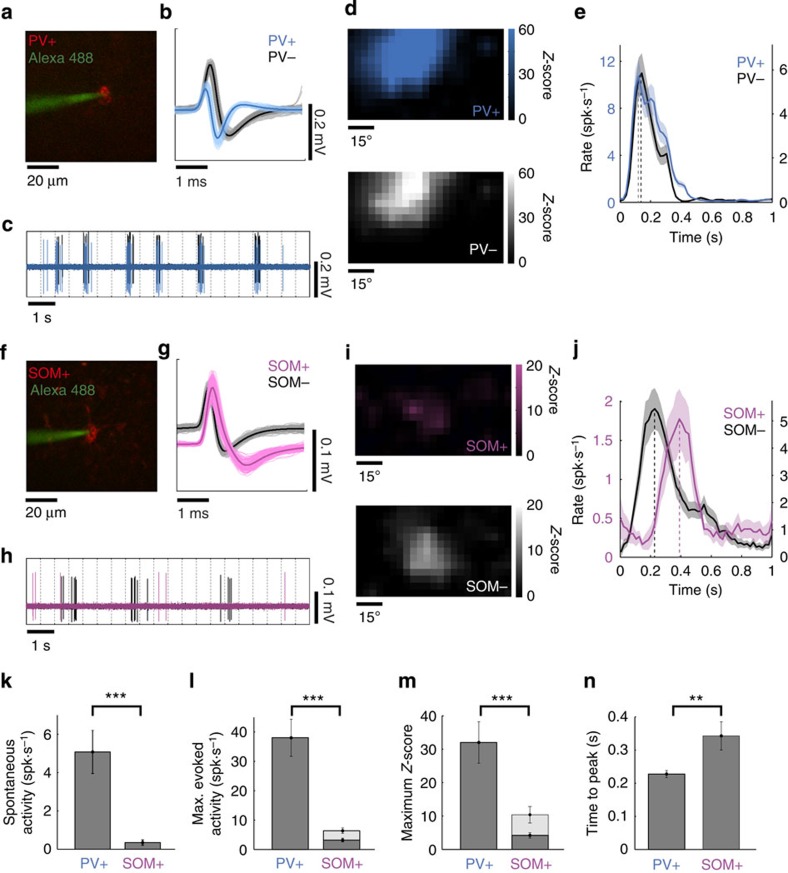
PV+ and SOM+ neuron responses to sparse noise stimuli. (**a**) *In vivo* targeted cell-attached recording of a PV+ neuron expressing ChR2-mCherry, using a glass pipette filled with Alexa 488. (**b**) Spike waveforms of the PV+ neuron recorded in **a** (blue) and of a putative pyramidal neuron recorded nearby (grey). Average waveforms are shown in dark colours. (**c**) High-pass filtered cell-attached recordings of the two neurons in response to the same sequence of sparse noise. Dashed lines indicate new stimulus onsets. (**d**) Receptive fields for ON stimuli in *Z*-score values for the PV+ neuron (top) and the pyramidal neuron (bottom). (**e**) Averaged evoked PSTHs in response to the complete sparse noise sequence for the PV+ neuron (blue) and the pyramidal neuron (gray). Respective scales are shown on both sides of the graph. S.e.m. values are shown as shaded areas. (**f**–**j**) Same as **a**–**e** for a SOM+ neuron (pink) and a putative pyramidal neuron (grey). (**k**–**n**) Population comparison of spontaneous activity (**k**, ***P<0.001, two-tailed *t*-test), maximum evoked activity (**l**, ***P<0.001, two-tailed *t*-test), maximum *Z*-score (**m**, ***P<0.001, two-tailed *t*-test) and response time-to-peak (**n**, **P=0.005, two-tailed *t*-test) between PV+ (*n*=22) and SOM+ (*n*=37) neurons. Cells without significant evoked responses (Z-score<3) were not included in the comparison of time-to-peak response (which thus included *n*=22 PV+ neurons and *n*=16 SOM+ neurons). Error bars indicate s.e.m. The grey bars in **l** and **m,** respectively, indicate the maximum firing rate and *Z*-score computed over all SOM+ neurons that displayed significant responses.

We next directly assessed the timing and amplitude of PV+ and SOM+ neuron responses to both sparse noise and full-field stimuli using two-photon targeted, cell-attached recordings. We used the CreLoxP recombination system to express mCherry-channelrhodopsin-2 (ChR2) in specific cell types using PV-Cre and SOM-Cre mouse lines (Supplementary Fig. 5). Expression of mCherry allowed us to target these inhibitory neuron classes using glass pipettes filled with the dye Alexa 488 (Fig. 2a,f). Successful patch recordings could be assessed by rapid and reliable spiking evoked in the patched neuron by activating ChR2 with blue light pulses and by *post hoc* identification of the spike waveform as fast-spiking (PV+ neurons) or regular-spiking (SOM+ neurons) (Fig. 2b,g and Supplementary Fig. 6).

PV+ neurons (*n*=22 neurons, 8 animals) responded strongly to sparse noise stimuli and displayed receptive fields that were comparable to those observed for putative pyramidal cells (Fig. 1), as also illustrated by cell-attached recordings of adjacent putative pyramidal cells performed during the same experiment (Fig. 2b–e). The firing pattern of PV+ neurons and pyramidal neurons evoked by the same sequence of sparse noise displayed strong similarity in their spatiotemporal receptive fields properties (Fig. 2c–e). In contrast, the firing pattern of SOM+ cells during sparse noise stimulation was distinct from the firing pattern of adjacent pyramidal cells (Fig. 2f–j); SOM+ neurons did not share much similarity in their response magnitude or time-to-peak response (Fig. 2i,j). Overall, compared with PV+ neurons, SOM+ neurons had very low spontaneous and evoked firing rates in response to sparse noise (Fig. 2k,l). Many of the recorded cells (21 out of 37 neurons, 8 animals) did not display significant responses to the sparse noise. The remaining cells (*n*=16) displayed weak responses (Fig. 2m) that were significantly delayed^33^ with respect to PV+ or pyramidal cells (Fig. 2j,n).

**Figure 3:**
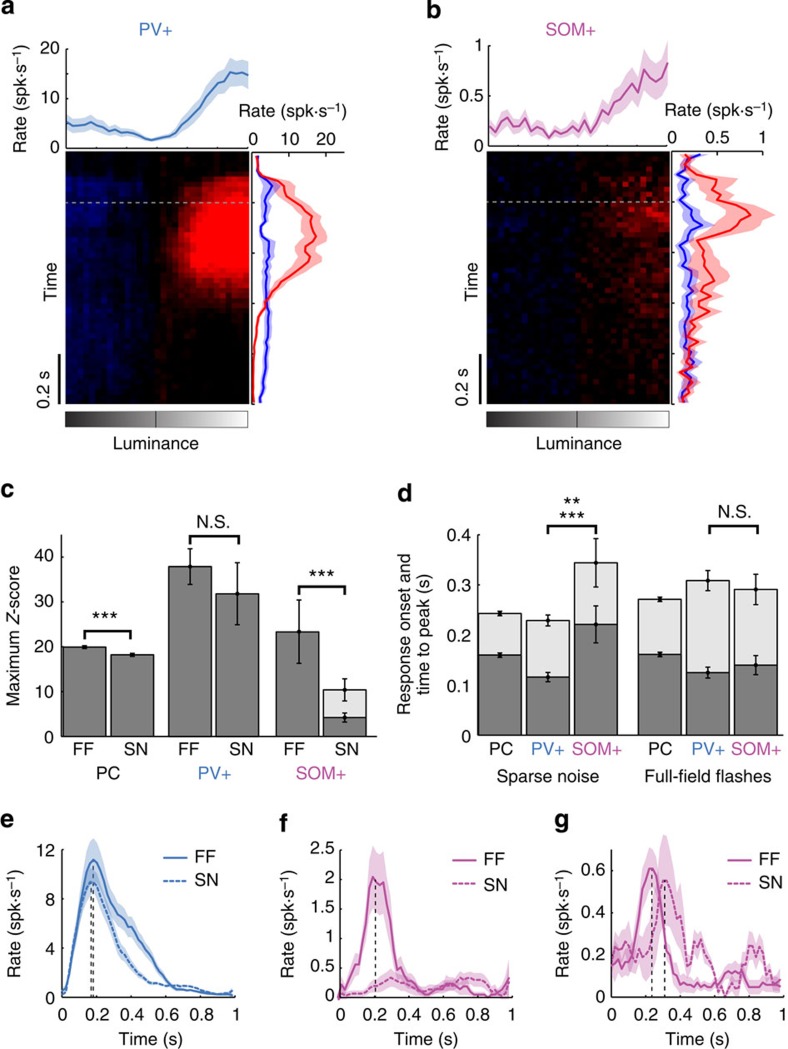
PV+ and SOM+ neuron responses to full-field flashes. (**a**) Targeted cell-attached recordings of PV+ neuron (*n*=20) responses to full-field flashes of various luminance intensities as a function of time. The curves on the right margin indicate the average response time course for ON (red) or OFF (blue) stimuli. The intensity curve on top is the estimated rate averaged around the dashed grey line (200 ms±100 ms). (**b**) Same as **a** obtained by targeted cell-attached recordings of SOM+ neurons (*n*=23). (**c**) Bar plots comparing the maximum *Z*-score of pyramidal (PC), PV+ and SOM+ neurons in response to sparse noise (SN) and full-field flashes (FF). PC: *n*=1,928 for SN and *n*=1,518 for FF. PV+ neurons: *n*=22 for SN and *n*=20 for FF. SOM+ neurons: *n*=37 for SN and *n*=23 for FF. For SOM+ neurons, the grey bar indicates the maximum *Z*-score computed over all SOM+ neurons that displayed significant responses during sparse noise (*n*=16). ***P<0.001 (two-tailed *t*-test) comparing responses for all cells during sparse noise and full-field flashes; NS, not significant, *P*=0.43. (**d**) Bar plots comparing the response onset (grey bars) and time-to-peak (dark bars) of pyramidal cells (PC), PV+ and SOM+ neurons in response to sparse noise (SN) and full-field flashes (FF). PC: *n*=761 for SN and *n*=620 for FF. PV+ neurons: *n*=22 for SN and *n*=20 for FF. SOM+ neurons: *n*=16 for SN and *n*=21 for FF. **P=0.005 for peak and ***P<0.001 for onset response (two-tailed *t*-test); NS, not significant, *P*=0.41 for peak and *P*=0.4 for onset response. (**e**) Averaged evoked PSTHs in response to sparse noise (dashed line) or full-field (continuous line) for a PV+ neuron. Response time-to-peak for each curve is indicated with a vertical dashed line. (**f**) Averaged evoked PSTHs in response to sparse noise (dashed line) or full-field (continuous line) for a SOM+ neuron that displayed a change in *Z*-score amplitude. (**g**) Same as **f** for a SOM+ neuron that displayed a change in response onset. Error bars and shaded areas indicate s.e.m. throughout the figure.

The functional properties of PV+ and SOM+ neurons were also investigated with full-field flashes. PV+ neurons (*n*=20 neurons, 4 animals) displayed monotonically increasing responses with contrast for ON and OFF stimuli, with a much weaker response for OFF stimuli (Fig. 3a) similar to the responses found for putative pyramidal cells (Fig. 1f). For these large stimuli, SOM+ neurons responded with a nearly identical monotonic intensity response curve (Fig. 3b). The maximum *Z*-scores computed for these neurons were significantly higher for full-field flashes compared with sparse noise (Fig. 3c). Virtually all SOM+ neurons (21 out of 23 neurons, 8 animals) responded significantly to full-field flashes. Moreover, they responded with significantly shorter response time-to-peak and onset time (defined as the time at which the *Z*-score first exceeded the significance threshold, see Methods), comparable to those of pyramidal or PV+ neurons (Fig. 3d, *n*=620 for putative pyramidal cells, *n*=20 for PV+ neurons and *n*=21 for SOM+ neurons). The changes in response strength, onset time and time-to-peak were evident in example PV+ (Fig. 3e) and SOM+ neurons (Fig. 3f,g). These data thus demonstrate that SOM+ neurons display very different response modes to the two stimuli used here: when sparse noise stimuli are presented, they have weaker responses at longer latencies than pyramidal and PV+ neurons, whereas, when full-field flashes are presented, they have stronger responses at latencies compared with pyramidal and PV+ neurons. These stimuli represent extremes of a continuum related to spatial summation by SOM+ cells^34^, and thus enable us to analyse how their response modes relate to their function.

### Stimulus-dependent inhibition by PV+ and SOM+ neurons

On the basis of the distinct response timings of PV+ and SOM+ neurons, we sought to understand how these differences translate to the interaction of PV+ and SOM+ neurons with local pyramidal cells during processing of sparse noise or full-field stimuli. We first ensured that we could evoke spikes from ChR2-expressing PV+ and SOM+ neurons precisely in time. Using targeted cell-attached recording, we applied single pulses of blue light (10 ms duration) that evoked reliable spiking responses in PV+ or SOM+ neurons with millisecond precision during concurrent visual stimulation; importantly, the ChR2-evoked spikes were similar across visual stimulation conditions and were independent of visual responses (Supplementary Fig. 7). Our goal was to reliably elicit moderate inhibition of visual responses in target cells, without evoking network disinhibition^25^,^39^,^40^ or circuit-wide changes^41^, so that we could compare effects across different types of inhibitory neurons and large numbers of target cells.

**Figure 4:**
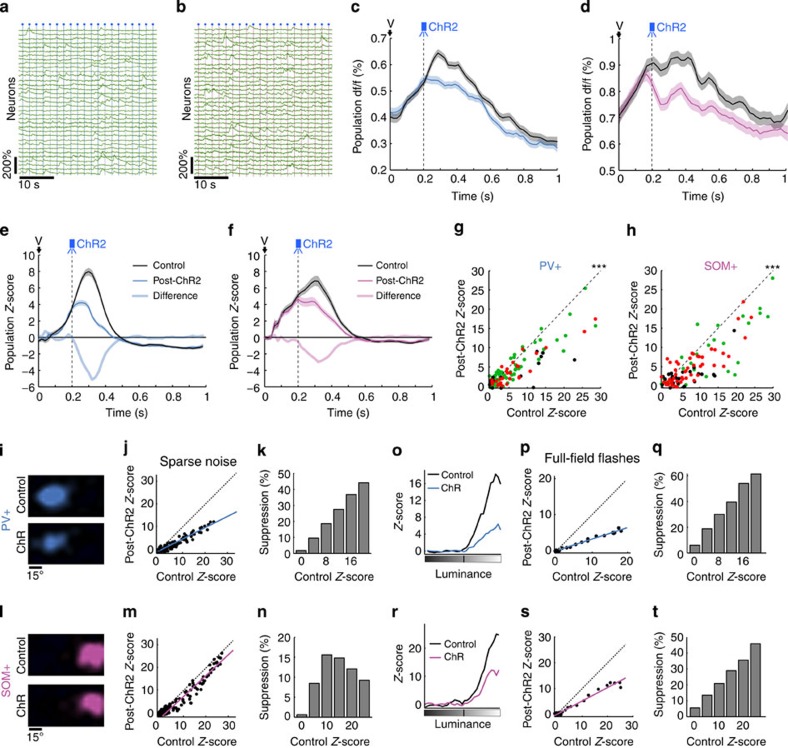
Single-pulse ChR2 stimulation of PV+ and SOM+ neurons reveals response dependence of inhibition. (**a**,**b**) Calcium traces corresponding to neuron responses to full-field flashes with blue light pulses delivered every two trials 0.2 s after stimulus onset (blue dots) for ChR2 excitation of (**a**) PV+ or (**b**) SOM+ neurons. (**c**,**d**) Population *df*/*f* averaged over all neurons in **a** and **b**, respectively, and over all trials in absence (black) or presence (blue/pink) of blue light pulses. Onset times of the visual stimulus (V) and ChR2 pulse (blue light) are indicated. S.e.m. valuesare shown with shaded areas. (**e**,**f**) Population activity time course after *df*/*f* deconvolution in the control condition (black) and during PV+ (**e**, blue) or SOM+ (**f**, pink) neuron stimulation. Difference between responses in these two conditions is shown in light colours. (**g**,**h**) Results of three experiments (different colors), comparing the average response of neurons in the control condition and with ChR2-stimulation of PV+ (*n*=113, *P*<0.001, Wilcoxon signed-rank test) or SOM+ neurons (*n*=104, *P*<0.001, Wilcoxon signed-rank test). (**i**) Receptive fields of a neuron imaged during control condition (top) or PV+ activation (bottom). (**j**) Point-by-point comparison between responses at corresponding receptive field locations shown in **i** (linear fit: slope=0.5, r=0.93, *P*<0.001, paired *t*-test). (**k**) Suppression strength as a function of the *Z*-score in the control condition for the receptive field shown in **i**. Values on the *x*-axis indicate the centre of bars and the bin size is 4. (**l**–**n**) Same as **i**–**k** but during ChR2 stimulation of SOM+ neurons. (**m**) Slope=0.97, *r*=0.94, *P*<0.001, paired *t*-test. The bin size in **n** is 5. (**o**) Luminance-response curves of a neuron imaged in the control condition (black) and during PV+ (blue) neuron stimulation. (**p**) Point-by-point comparison for each luminance between control and PV+ activation conditions corresponding to the curves in **o** (linear fit: slope=0.36, *r*=0.99, *P*<0.001, paired *t*-test). (**q**) Suppression strength as a function of the *Z*-score in the control condition (bin size=4) for the curves shown in **o**. (**r**–**t**) Same as **o**–**q** but during SOM+ neuron activation. Line in (**s**), slope=0.49, *r*=0.99, *P*<0.001, paired *t*-test. The bin size in **t** is 5.

We then studied the suppression strength of the evoked response by systematically varying the timing of the 10-ms long ChR2 pulse between 0.1 s before stimulus onset to beyond the response peak after visual stimulus onset and found that a near-optimal time to activate inhibitory neurons by the ChR2 pulse was at or just before 0.2 s following visual stimulation onset (Supplementary Fig. 8). This timing falls on the rising phase between the onset and the peak amplitude of visually evoked responses in typical pyramidal cells (Fig. 4a–f). This suppressive effect was evident at the level of individual cells when comparing their control response amplitude with that following the light pulse (Fig. 4g,h, *n*=113 for PV+ and *n*=104 for SOM+ neurons); the vast majority of cells showed suppression, with very few cells displaying facilitation that would reflect a disinhibitory mechanism. We also performed control experiments in wild-type mice confirming that the effect we observed was exclusively due to ChR2 activation and not caused by other uncontrolled sources (Supplementary Fig. 9). Thus, stimulating either PV+ or SOM+ neurons with single pulses in effect results in a compound IPSC in postsynaptic neurons, leading to direct suppression of visually evoked responses.

We then measured neuron responses and receptive fields using sparse noise visual stimuli, in the presence or absence of single blue light pulses to stimulate PV+ or SOM+ neuron populations (Fig. 4i–n). To assess the effect of specific inhibitory neuron classes, we compared point-by-point responses from the spatial receptive field obtained during ChR2-activation of these neurons with the control receptive field (Fig. 4i,l). When PV+ neurons were stimulated, the effect on the target receptive field structure was divisive, as shown by the linear relation between control and post-ChR2 responses with a slope <1 (Fig. 4j). That is, we observed stronger suppression for strongly responsive parts of the receptive field and weaker suppression for regions away from the centre of the receptive field, so that the suppression strength was strongly dependent on the control response (Fig. 4k)—indicating divisive inhibition. In contrast, the suppression evoked by SOM+ neurons rather uniformly affected the target neuron’s responses and receptive field (Fig. 4l), shifting all responses uniformly downwards and resulting in a linear relationship between suppression strength and control response with a slope close to 1 (Fig. 4m)—indicating response-independent subtractive inhibition (Fig. 4n). Other single-cell examples of these distinct suppression patterns are shown in Supplementary Fig. 10.

The suppression patterns of PV+ and SOM+ neurons suggest a network origin of their functions that could potentially be altered with full-field stimuli. Indeed, when we used full-field flashes to probe the properties of PV+ and SOM+ mediated inhibition, we obtained very different effects for SOM+ neurons. By comparing, for each intensity, the luminance response curve of individual pyramidal cells in the control condition with the response when specific inhibitory neurons were stimulated (Fig. 4o,r), we could assess the nature of inhibition evoked by these cell types. While PV+ neurons still showed divisive effects on pyramidal neuron responses (Fig. 4p,q), SOM+ neurons now displayed divisive effects nearly identical to PV+ neurons (Fig. 4s,t). Thus, both PV+ and SOM+ neurons can provide divisive inhibition in response to a stimulus that strongly drives both neuron types along with their target cells, at comparable visual latencies. In response to sparse noise stimuli, however, which drive pyramidal and PV+ neurons effectively at short latencies but not SOM+ neurons, PV+ and SOM+ neurons perform different operations on target pyramidal cells (see Supplementary Fig. 10 for more single-cell examples).

**Figure 5:**
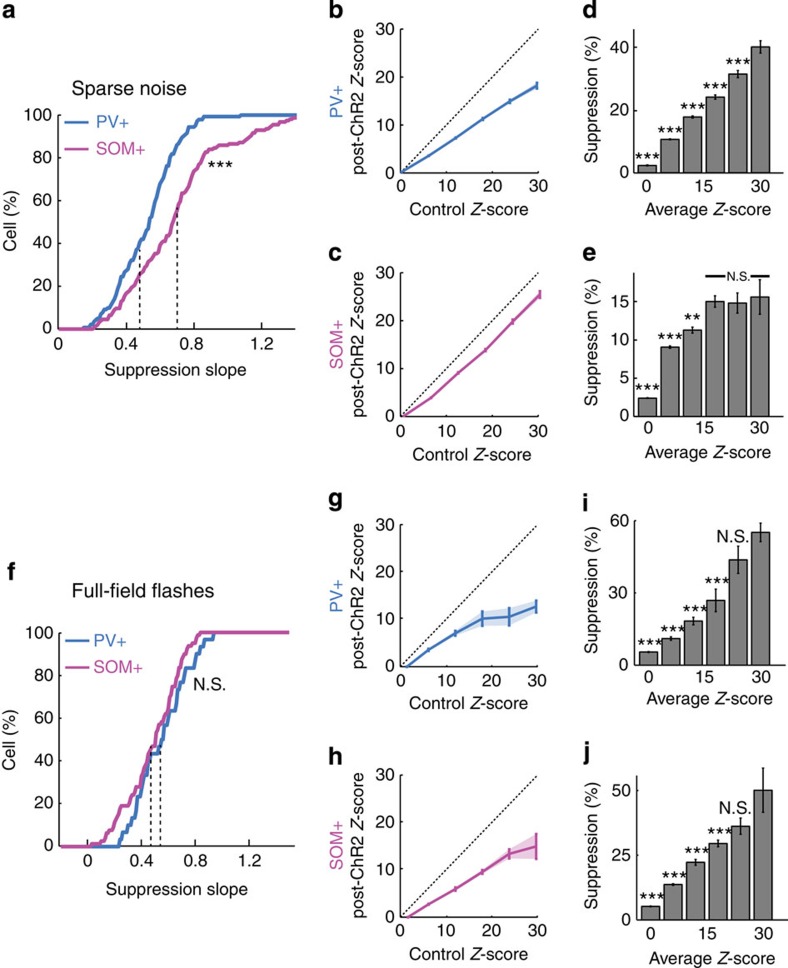
Population analysis of response modulation during activation of PV+ and SOM+ neurons. (**a**) Cumulative distribution of the linear fit slope for all cells that displayed a significant suppression (*n*=87 for PV+ and *n*=119 for SOM+ neurons, *P*<0.01, Wilcoxon signed-rank test) in response to PV+ (blue) or SOM+ (pink) activation during sparse noise presentation (*P*<0.001, unpaired *t*-test). Average values are indicated by dashed lines. (**b**,**c**) Comparison of the population *Z*-score averaged over all neurons, between the control condition and when PV+ (**b**) or SOM+ (**c**) neurons were stimulated. The blue and pink lines indicate the average over all neurons (blue slope=0.63; pink slope=0.91), bars indicate s.e.m. (**d**,**e**) Histogram of the suppression strength for different ranges of baseline *Z*-scores, averaged over all neurons for PV+ (**d**) and SOM+ (**e**) neurons. (**b**,**d**) From left to right: *n*=20,914, 3,449, 1,065, 358, 141, 56 data points. (**c**,**e**) From left to right: *N*=28,598, 4,158, 1,475, 674, 344, 175 data points. Error bars indicate s.e.m. and bin size for each bar is 6. All statistical tests were done in comparison with the maximum *Z*-score range: **P<0.01; ***P<0.001; NS, not significant, unpaired *t*-test. (**f**–**j**) Same as **a**–**e** but for full-field flashes and during stimulation of PV+ or SOM+ neurons. (**f**) *n*=30 for PV+ and *n*=79 for SOM+ neurons. NS not significant, unpaired *t*-test, *P*=0.12. (**g**) Slope=0.44 and (**h**) Slope=0.55. (**g**,**i**) From left to right: *N*=614, 140, 56, 10, 5, 12 data points. (**h**,**j**) From left to right: *N*=1,539, 437, 118, 79, 27, 6 data points. (**g**–**j**) Error bars indicate s.e.m.

We confirmed these results at the population level. For all visually responsive neurons that displayed a significant (but not complete) suppression in response to PV+ or SOM+ neuron stimulation during the sparse noise protocol, the distribution of the ‘suppression slope’, or slope of the line relating post-ChR2 response and control response (for example, Fig. 4j,m), was significantly lower for PV+ neurons than for SOM+ neurons (Fig. 5a, *n*=87 neurons from 6 animals for PV+, *n*=119 neurons from 6 animals for SOM+). It is also worth mentioning that, although these two distributions are significantly different, the effect of PV+ and SOM+ activation is not exclusively divisive or subtractive. Indeed, suppression slopes much smaller than 1 were observed for a small proportion of cells that were strongly suppressed by SOM+ activation (Supplementary Fig. 11). Interestingly, these target cells also had long response latencies to sparse noise stimuli (Supplementary Fig. 11e), comparable to SOM+ latencies (Fig. 3d), indicating that rather than being an exception, these cells were consistent with the hypothesis that the temporal overlap of SOM+ and target responses shapes the effect of SOM+ inhibition. Pooling cells from individual experiments and computing the average *Z*-score change (between control and ChR2-stimulation) and the s.e.m. in sparse noise experiments nevertheless demonstrated low variability for the effects of PV+ (Fig. 5b, *n*=87 neurons from 15 experiments, 6 animals) and SOM+ (Fig. 5c, *n*=119 neurons from 15 experiments, 6 animals) neuron activation. Analysis of the average suppression as a function of control *Z*-score showed that, while PV+ neurons caused a significant scaling of suppression with control response (Fig. 5d), SOM+ neurons showed no dependence on the stimulus strength for large control values (Fig. 5e; the weaker suppression observed for low *Z*-scores can be attributed to a floor effect: responses that are close to the spike threshold cannot be suppressed below the threshold, resulting in weaker suppression). Strikingly, the same population analyses applied to full-field flashes did not show any significant differences between PV+ and SOM+ neurons (Fig. 5f–j, PV+: *n*=30 neurons from 8 experiments, 3 animals; SOM+: *n*=79 neurons from 15 experiments, 4 animals). One-way ANOVA revealed a significant difference between the four groups in Fig. 5a,f (*P*<0.001 with Bonferroni correction for multiple comparisons). Using *post-hoc* pairwise *t*-tests, we found that only SOM+ activation in the sparse noise condition was significantly different from all the other groups.

**Figure 6:**
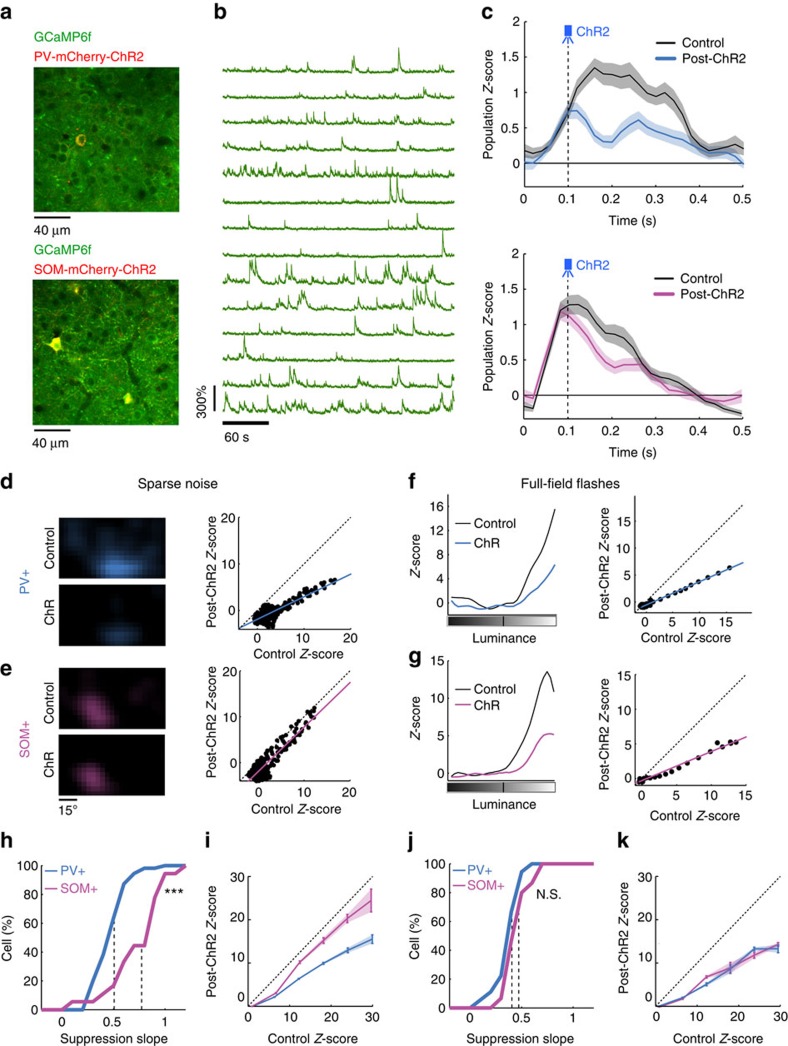
Effect of ChR2-mediated PV+ and SOM+ neuron activation on target cells of awake head-fixed mice. (**a**) Image of a region of V1 expressing GCaMP6f in all neurons and mCherry-ChR2 in PV+ (top) or SOM+ (bottom) neurons. (**b**) Example calcium traces normalized to baseline (*df*/*f*) obtained during presentation of sparse noise. (**c**) Population responses in control condition (black) and when a blue light pulse was delivered to PV+ (blue, *n*=30) or SOM+ (pink, *n*=26). In awake mice the responses were faster than in the anaesthetized case and we therefore used pulses at 0.1 s instead of 0.2 s. S.e.m. values are shown as shaded areas. (**d**) Left: example receptive fields of a neuron imaged during control condition (top) and during PV+ activation (bottom). Right: point-by-point comparison between responses at corresponding receptive field locations. The blue line indicates the linear fit (slope=0.47, *r*=0.93, *P*<0.001, paired *t*-test). (**e**) Same as **d** but during ChR2-stimulation of SOM+ neurons. Slope=0.93, *r*=0.88, *P*<0.001, paired *t*-test. (**f**) Left: example luminance-response curves of a neuron imaged in the control condition (black) and during PV+ (blue) neuron stimulation. Right: point-by-point response comparison for each luminance. The blue line indicates the linear fit (slope=0.44, *r*=1, *P*<0.001, paired *t*-test). (**g**) Same as **f** but during SOM+ neuron activation. Slope=0.42, *r*=0.99, *P*<0.001, paired *t*-test. (**h**) Cumulative distributions of the linear fit slope for all cells that displayed a significant suppression (*P*<0.01, Wilcoxon signed-rank test) in response to PV+ (blue) or SOM+ (pink) activation during sparse noise presentation (*n*=55 for PV+ and *n*=18 for SOM+, *P*<0.001, unpaired *t*-test). (**i**) Comparison of the population *Z*-score averaged over all neurons, between the control condition and when PV+ (*n*=55, slope=0.56) or SOM+ (*n*=18, slope=0.89) neurons were stimulated during sparse noise. (**j**–**k**) Same as **h**–**i** but during full field-flashes. (**j**) *n*=18 for PV+ and *n*=15 for SOM+, *P*=0.11. (**k**) PV+ (*n*=18, slope=0.51) and SOM+ (*n*=15, slope=0.50). One-way ANOVA revealed a significant difference between the four groups in **h** and **j** (*P*<0.001 with Bonferroni correction for multiple comparisons). Using *post-hoc* pairwise *t*-tests, we found that only SOM+ activation in the SN condition was significantly different.

Several recent studies have shown that the level of inhibition is markedly higher in awake behaving mice^42^, in which SOM+ neurons in particular can display higher firing rates compared with anaesthetized conditions^34^,^43^,^44^. To examine the validity and generality of our results, we performed the same experiments in awake head-fixed mice using the genetically encoded calcium indicator GCaMP6f instead of OGB-1AM (see Methods). Our results were similar to the anaesthetized case (Fig. 6) indicating that, at least in awake non-behaving mice, the function of SOM+ neurons remains stimulus-dependent. However, we do not exclude the possibility that changes in brain state, such as during locomotion^43^,^45^, could alter the function of various inhibitory cell types.

### Stimulus-dependent inhibition in a recurrent network model

**Figure 7:**
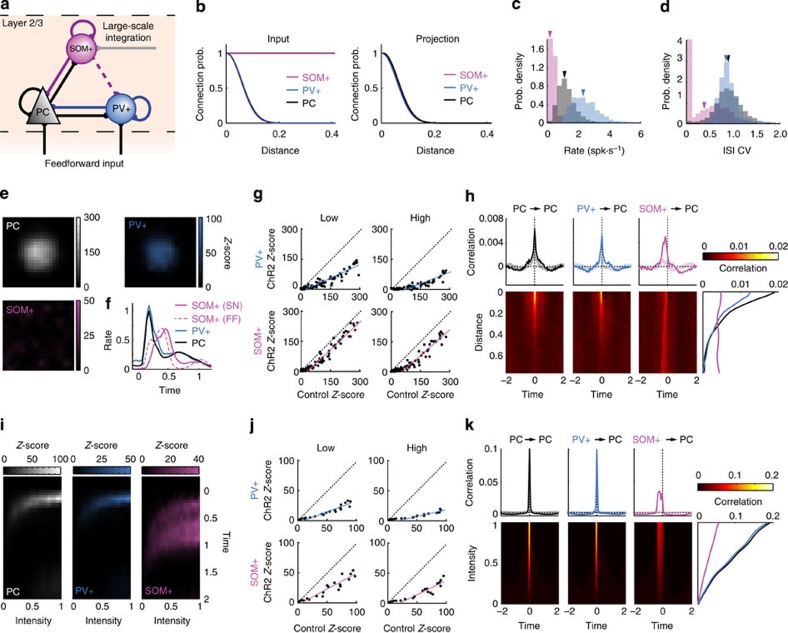
Network model of superficial layer V1 representing the connections and functions of SOM+, PV+ and pyramidal neurons. (**a**) Schematic of model connections. (**b**) Connection probability distribution for each cell type depicting inputs (left) and output projections (right). (**c**,**d**) Distribution of the spontaneous firing rate (**c**) and interspike interval coefficient of variation (ISI CV, **d**) for each cell type. Average values for each population are indicated with coloured arrows. (**e**) Receptive fields of each neuron population in the network in response to small sparse-noise stimuli. (**f**) Time course of spatially averaged responses obtained in **e**, normalized to the peak response of pyramidal cells for sparse noise. The dashed pink line shows the response of SOM+ neurons to full-field flashes normalized to the peak response of pyramidal cells in the same condition. The time scale is in units of stimulus duration. (**g**) Comparison between responses obtained in the control condition and when PV+ (top) or SOM+ (bottom) neuron populations were activated with a single pulse of excitation at ‘low’ (left) or ‘high’ (right) levels (PV+ cells: 20 and 30 nS; SOM+ cells: 40 and 50 nS). (**h**) (Bottom) Average pairwise correlation between pyramidal neurons (left), PV+ and pyramidal neurons (middle) and SOM+ and pyramidal neurons (right) as a function of time difference and distance in the network. The time scale is in units of stimulus duration. (Top) Correlation versus time difference, averaged across distance, for the various cell combinations. Light coloured lines indicate the baseline pairwise correlation during ongoing spontaneous activity; dark lines indicate correlations during visually driven activity. The panel on the right is the average correlation at time difference 0 as a function of distance. (**i**) Population responses during full-field stimulation as a function of increasing drive for pyramidal (left), PV+ (middle) and SOM+ (right) neurons. (**j**) Same as **g** for full-field flashes. (**k**) Same as **h** but as a function of drive intensity and time difference.

To mechanistically understand these experimental findings, we designed a simple network model^46^ of layer 2/3 in which the different cell types were randomly distributed in a two-dimensional layer. On the basis of the strong response similarity between pyramidal and PV+ neurons and the short delay of PV+ responses to small stimuli^33^, we modelled both these cell types as receiving direct feedforward inputs from a parallel two-dimensional layer depicting thalamorecipient layer 4 neurons (Fig. 7a) to model retinotopic receptive fields. The local recurrent connections of these cell types were sparse and followed a Gaussian distribution. In contrast, the weak and delayed responses of SOM+ neurons to sparse noise, as well as their lack of response during ChR2-stimulation of layer 4 excitatory cells *in vitro*^34^, suggested that SOM+ neurons do not receive feedforward sensory input but exclusively receive their excitatory drive from local excitatory neurons. We modelled these sparse connections with a uniform connection probability distribution mimicking the long-range interaction with local pyramidal cells^34^. All cell types projected locally according to a distant-dependent Gaussian distribution (Fig. 7b). We also added unidirectional connections from SOM+ to PV+ neurons to replicate intracortical connections^25^,^40^ and size tuning curves^34^, although these connections were not necessary to produce the effects described here (Supplementary Fig. 12). Synaptic interactions were modelled as instantaneous increases in excitatory conductances (from pyramidal neurons) or inhibitory conductances (equivalently from PV+ and SOM+ neurons) followed by exponential decays.

The spontaneous activity observed in our model was comparable to the spontaneous activity observed in our experiments *in vivo*, displaying low firing rate of SOM+ cells, relatively low firing rate of pyramidal cells and higher firing rate of PV+ cells (Fig. 7c) with highly irregular firing patterns^47^ (Fig. 7d) and weak distance-dependent correlation (Fig. 7h, light coloured lines) similar to our data (Supplementary Fig. 4i) and that of others^38^. The spatio-temporal correlation structure was dependent on several parameters of the model including the number of connections per neuron relative to the network size and typical synaptic strength^46^. We used a set of parameters that reproduced important features of the dynamics while keeping the network simple.

To model sparse noise stimuli, we applied a small transient Gaussian ‘visual’ stimulation pattern in layer 4 and analysed the activity of the network. Using the symmetry of the network (homogeneous connectivity pattern and periodic boundary conditions), receptive fields were obtained by computing the response maps of the network for each population. Because of their local connectivity and feed-forward input, pyramidal cells and PV+ cells displayed a localized and strong response to the small stimulus. In contrast, the lack of feedforward input and the large-scale integration of SOM+ neurons led to a delayed and spatially uniform but weaker response to the stimulus (Fig. 7e,f). (The absolute response latencies generated by the model were arbitrary, because retinal, retinofugal conduction and multiple synaptic latencies were not included.) Note also that SOM+ neuron response variability found in *in vivo* data (Fig. 3f,g) was not accounted for here since the network was symmetrical and therefore displayed a single average behaviour compared with the average experimental result. Adding some heterogeneity in the connectivity rule of SOM+ neurons could potentially reproduce the range of response profiles observed experimentally. Activating PV+ and SOM+ cells with a pulse of excitation (mimicking a ChR2 pulse) during ‘visual’ stimulation resulted in divisive inhibition by PV+ neurons and subtractive inhibition by SOM+ neurons (Fig. 7g). This was a consequence of the different sensitivities and delay of PV+ and SOM+ neurons to small, local stimuli. The SOM+ neuron population responded uniformly to small ‘visual’ stimuli with a significant delay compared with pyramidal neurons; when probed with a single ‘ChR2’ pulse, the inhibition elicited by this population was homogeneous across pyramidal cells (independent of location) and resulted in a subtractive effect. In contrast, the response of PV+ neurons to small visual stimuli was strongly dependent on their location in the network and closely resembled the response profile of nearby pyramidal cells. As a result, the inhibition evoked by these neurons in target cells was stronger for stronger responses (in the center of the receptive field) and resulted in a divisive effect. These effects were not dependent on the strength of stimulation and SOM+ neurons usually required a stronger pulse of activity to produce a significant suppression of pyramidal cells (as also observed experimentally). The stimulus dependence of the various cell types was further evident in their correlation with local pyramidal cells as a function of stimulus parameter (Fig. 7h). The correlation between pyramidal and PV+ cells produced by this stimulus decayed over distance, whereas it was uniform for SOM+ neurons.

Full-field flashes of increasing intensity were reproduced by transiently stimulating the entire network with increasing drive. In this case, the functional properties of all cell types were more comparable, showing a monotonic increase in their response to increasing drive (Fig. 7i) and decrease of SOM+ neuron response latencies compared with sparse noise (Fig. 7f). Stimulating PV+ or SOM+ neurons with a pulse of ‘ChR2’ during presentation of full-field flashes resulted in divisive inhibition from both neuron types (Fig. 7j). For this stimulus ensemble, the correlation structure with pyramidal cells showed that all neuron types were similarly sensitive to the stimulus parameters, displaying similar temporal correlations and increasing correlation with increasing intensity (Fig. 7k). For SOM+ neurons in particular, the switch between subtraction and division may thus be explained by their connections and their consequent stimulus-dependent temporal and spatial relationship to pyramidal neuron responses. Importantly, the effect of SOM+ activation on target pyramidal neurons did not depend on SOM+ to PV+ connections (Supplementary Fig. 12), indicating that this disinhibitory circuit is unlikely to play a role in the effect of SOM+ neurons on pyramidal cells *in vivo*^27^,^40^.

This simple computational model reproduced the phenomenology observed *in vivo* only by using different connectivity rules for PV+ and SOM+ neurons that were otherwise modelled identically. These connectivity rules were inspired by recently published work^34^ and were kept as simple as possible. We do not exclude the possibility that the nature of GABA receptors targeted by these different inhibitory neuron types may differ^1^ and could also participate in this mechanism, although how this would enable SOM+ neurons to switch their function remains to be clarified.

## Discussion

We have shown in this study that PV+ and SOM+ neurons, the two well-defined classes of cortical inhibitory neurons that target pyramidal neurons, have functional effects on their targets that depend on their response modes and the nature of visual stimuli. When probed with small stimuli, the responses of SOM+ neurons are delayed and often weak, and differ significantly from those of pyramidal and PV+ neurons. Under these conditions, when probed with single pulses of ChR2, SOM+ neurons provide subtractive inhibition. With the same stimuli, PV+ neurons are coactive with pyramidal neurons, with similar response latencies, and provide divisive inhibition. Strikingly, when large stimuli are used, both PV+ and SOM+ neurons display comparable response latencies as pyramidal neurons, and both provide divisive inhibition.

It is possible that the function of PV+ and SOM+ neurons also depend on the cortical layer. Our experiments were performed in superficial layers II/III of V1, but we do not exclude the existence of alternative mechanisms in deeper layers. Moreover, although we tried to target superficial layers during viral injections, it is likely that deeper layers were also transfected and therefore the effect observed in our data could in principle include the contribution of interneurons in these layers, which in turn could interact with superficial layers in distinct ways. However, our results were consistent across animals indicating that despite a possible difference in PV+ and SOM+ neurons distribution within the cortical column, their collective effect on target cells in superficial layers is robust and reliable.

Our work differs in important ways from previous studies that examined the role of inhibitory neuron classes^14^,^15^,^26^,^40^. First, these studies used prolonged full-field visual stimulation to drive neurons, which can obscure the temporal relationship between inhibitory and target neuron firing. Second, these studies used prolonged optogenetic stimulation of inhibitory neurons to evaluate their effect on target neurons; such stimulation is superimposed on visual drive to neurons, and alters the magnitude and timing of visually evoked responses in inhibitory neurons and their targets. Moreover, such stimulation invokes not only inhibitory but also disinhibitory interactions in cortical networks^25^,^40^ as reflected by the stronger suppression evoked in pyramidal neurons by SOM+ neurons in our study using single pulse stimulation compared with prolonged pulse trains^40^. Prolonged ChR2 excitation can also result in depolarization block in neurons^48^. By employing briefly flashed visual stimuli, specific stimulus ensembles, and short pulses of optogenetic activation, we were able to define the respective response latencies of PV+ and SOM+ neurons and compare them with pyramidal neurons, as well as isolate the inhibitory effect of these neuron classes on their targets.

Our findings help explain the disagreement in previous reports on the functional role of PV+ and SOM+ neurons^14^,^15^,^26^. PV+ neurons are easily excited by ChR2 stimulation and they exert powerful feedforward inhibition: the disagreement regarding PV+ neurons is well explained by considering the intensity of PV+ activation in these studies^27^,^28^,^49^. In the case of SOM+ neurons, Lee *et al*.^15^ activated SOM+ cells continuously with prolonged ChR2 stimulation, and the visual stimulation was displayed full-field and completely contained within the ChR2 activation period. We hypothesize that the temporal overlap of pyramidal cell visual activation and SOM+ visual plus ChR2 activation fulfilled the condition for divisive inhibition. We have confirmed this idea by performing additional experiments with full-field drifting gratings and optogenetic pulse activation at the timing of onset response and found divisive inhibition for both PV+ and SOM+ neurons (Supplementary Fig. 13a,b). Wilson *et al*.^14^ used ChR2 activation only during the first second of the full-field visual stimulation, and probed the effect of inhibition subsequently, during which we hypothesize there was no temporal overlap between pyramidal and SOM+ responses, and hence found subtractive inhibition. This was further verified by performing another set of experiments where the ChR2 activation pulse was delivered in a later time (corresponding to the last stimulation pulse in ref. 14) when the visually evoked response of SOM+ neurons was almost back to baseline. In this protocol, the effect produced by PV+ or SOM+ neuron ChR2 activation was significantly different, with more subtractive inhibition elicited by SOM+ neurons (Supplementary Figs 13a,c and 14a,b). Furthermore, Lee *et al*.^27^ replicated results from Wilson *et al*.^14^ and showed that under different stimulation conditions SOM+ neurons can indeed carry out very different functions (Supplementary Fig. 14c–e). Our findings now demonstrate that the mechanistic basis for this functional switch is the response mode and timing of SOM+ neurons relative to their target cells.

The dynamic switch of neural computations performed by SOM+ neurons provides a basis for context-dependent switching of function. Most SOM+ neurons are insensitive to small stimuli or respond with a significant delay, and therefore evoke stimulus-independent suppression of local pyramidal cells in this sensory context or when activated by other inputs such as cholinergic drive^50^ or top-down modulation^51^. This subtractive inhibition can enhance the decoding properties of the cortical network by sharpening the tuning curves or receptive fields of pyramidal cells^13^,^14^. SOM+ neurons can switch to providing divisive inhibition and gain control when activated by large stimuli. In contrast, PV+ neurons mediate gain control for both small and large stimuli by uniformly evoking strong divisive inhibition in neighbouring pyramidal cells. The dynamics of SOM+ neurons to different sensory stimuli provides an additional gain control mechanism when large stimuli are processed. PV+ and SOM+ cells could then provide complementary functions, respectively, controlling the gain of pyramidal cells in the face of changes in local contrast or overall brightness when presented with scale-invariant stimuli such as natural scenes.

Furthermore, SOM+ neurons provide a way to dynamically integrate top-down inputs with sensory inputs: top-down inputs that activate SOM+ neurons in a temporally distinct manner from visual (bottom-up) inputs would help shape response selectivity, whereas top-down activation that temporally overlaps visual activation would provide gain control. As SOM+ neurons are particularly sensitive to brain states^43^,^51^, their dynamic response modes provide a rich way to regulate pyramidal cell responses.

## Methods

### Animals and virus injections

Experiments were carried out in mice under protocols approved by MIT’s Animal Care and Use Committee and conformed to NIH guidelines. Heterozygous SOM-Cre and PV-Cre knockin driver mice (Jackson Labs) were backcrossed into a C57BL/6 line and housed 1–5 mice per cage under a 12/12-h light/dark cycle. Adult mice (>6–8 weeks old, both genders) were anaesthetized with 4% isoflurane in oxygen. The skull was thinned at a location 3.5 mm posterior and 3 mm lateral to bregma, corresponding to the caudolateral part of V1. A glass micropipette filled with the virus (pAAV-Ef1a-DIO-hChR2(H134R)-mCherry-WPRE-pA from University of North Carolina viral vector core facility) was then inserted in the cortex through the remaining skull and the dura. Two injections of 250 nl were made, respectively, at a depth of 500 and 250 μm below the cortical surface at a rate of 100 nl min^−1^. Immunohistochemistry was performed to ensure the expression of the virus as previously described^14^ (Supplementary Fig. 5).

### Animal preparation

*In vivo* experiments were performed on mice 2 weeks or more post-injection. Mice were first anaesthetized with 1.5% isoflurane in oxygen and subsequently injected with a cocktail containing fentanyl (0.05 mg kg^−1^), midazolam (5 mg kg^−1^) and medetomidine (0.5 mg kg^−1^), supplemented with 0.5% isoflurane. Eyes were protected with ophthalmic ointment. A metal plate was attached to the skull using opaque black superglue and dental acrylic and screwed into a moveable stage. A circular craniotomy (1.5 mm diameter) centred on the injection site was then performed. A thin layer of 3% agarose in ACSF was used to cover the cortex. The stage was then transferred to the microscope where another tube was installed to deliver isoflurane. Throughout the surgery and the entire experiment, the body temperature was maintained at 37.5°C with a heating blanket (Harvard Apparatus) and if necessary additional heating pads.

### *In vivo*-targeted cell-attached recordings

To perform targeted cell-attached recordings, a borosilicate pipette back-filled with 4 μl of either Alexa 488 or Alexa 594 (Molecular Probes) was inserted into the brain at a 30° angle below a × 25 Olympus water immersion objective. The objective as well as the glass pipette and an Ag/AgCl ground pellet electrode were immersed in ACSF. The glass pipettes (outer diameter=1.5 mm, inner diameter=1.17 mm) were pulled using a Sutter P-1000 puller to obtain a baseline resistance of 3–7 MΩ. Recordings were made using custom software (Network Prism, Sur Lab) written in Matlab (Mathworks, Natick, MA) controlling a MultiClamp 700B Amplifier (Axon Instruments). The pipette was then advanced diagonally towards specific labelled neurons under the guidance of two-photon imaging at 910 nm and with an applied pressure of 15–30 mbar. Small lateral movements of few tens of microns were used to better reach targeted neurons. Cell approach was assessed visually and with a significant increase of resistance (>twofold). After the cell was successfully targeted, pressure was released creating a strong increase in resistance (20–100 MΩ). Weak negative pressure was applied to hold the cell (about −10 mbar). For targeted cells that expressed the light-gated ion channel channelrhodopsin (ChR2), we could confirm successful cell-attached patch by triggering spikes with blue light (see below) at 1-ms precision. To ensure that cells with very low firing were still recorded during long recording sessions, we regularly evoked spiking activity in them via ChR2 by applying pulses of blue light. The amplifier was switched to current clamp mode and spikes were recorded with zero injected current under a Bessel filter of 4 kHz and an AC filter of 300 Hz.

### Two-photon calcium imaging in anaesthetized mice

A solution containing 50 μg of Oregon Green BAPTA 1-AM (Invitrogen) mixed with 5 μl of pluronic acid (20% in DMSO) wrapped in aluminum foil was centrifuged for 20 min and subsequently mixed with 45 μl of a solution containing 2 μl of Alexa 488 (Invitrogen) diluted in 200 μl of saline. This mixture was then centrifuged through a 0.22 μm filter (Millipore) for 30 s and stored on ice. Glass pipettes identical to the ones used for targeted cell-attached recording were back-filled with 3 μl of the dye. The pipette was then inserted in the cortex under two-photon guidance to the region of interest (virus injection site) where mCherry-ChR2 was expressed in either PV+ or SOM+ neurons. In experiments where no optogenetic manipulations were necessary, we aimed at region of the cortex free of large blood vessels. Once in position, sustained but light positive pressure was incrementally applied using a Picospritzer II until the dye started to leak out of the pipette with a slow and constant flow. The loading was maintained for about a minute. The dye uptake usually enabled imaging 30–60 min after bulk loading.

Imaging was performed with a modified Prairie Ultima two-photon system (Prairie Technologies) driven by a Spectra Physics Mai-Tai eHP laser. All functional imaging was done using a × 25 Olympus XL Plan N high-numerical index objective lens. The details of the system have been described in detail previously^14^. In brief, a custom all-Matlab system (Network Visor, Sur Lab) was used to detect changes in fluorescence. The red and green PMT cables from the two-photon microscope were forked and fed into a custom A/D acquisition system and graphical user interface (National Instruments and Matlab). The control lines of the two-photon’s X and Y galvanometers were in turn substituted with D/A control lines from the custom system. After an initial raster scan that provided an image of the OGB-loaded cells, their positions were detected to establish a scan-path. Using a genetic algorithm, the shortest scan-path between cells was then computed and the imaging was performed along this trajectory. This strategy could yield calcium traces of about a hundred cells imaged sequentially at a 50 Hz sampling rate and high signal-to-noise ratio. All imaging sessions had a duration of 2 min. In between each session, an image of the cortex was taken to ensure that the scan-path was still aligned with the cells. If a movement occurred during a session, the cortex was realigned on the scan-path and the session was discarded.

### Two-photon imaging in awake head-fixed mice

We performed calcium imaging experiments in awake head-fixed PV-Cre or SOM-Cre mice expressing GCaMP6f (ref. 52) (UPenn Vector Core: AAV1.Syn.GCaMP6f.WPRE.SV40) in all neurons and mCherry-ChR2 in a Cre-dependent manner. A craniotomy was performed before virus injection and the cortex was then protected by a 3-mm glass coverslip further sealed with Metabond permanent cement mixed with black ink to ensure opacity. A custom-made headplate was implanted on the mouse skull and attached with Metabond permanent cement. After a week-long recovery period, the mouse was habituated to head-fixation by screwing the headplate to an immobile stage and confining the mouse in a tube. Imaging experiments were done at least 2 weeks after virus injection. We performed the same experiments that were done in anaesthetized state except that we used Scanimage (HHMI Janelia Farm) to acquire full frame images with a frame rate of 25 Hz, with high zoom and low resolution so that roughly 25 well-isolated cells could be imaged simultaneously. Movies were movement-corrected before any further analysis.

### Optogenetic stimulation protocol

Neurons expressing channelrhodopsin were driven by a diode-pumped solid state blue laser with analogue intensity control (473 nm, 200 mW, MBL-III-473, OptoEngine, LLC) coupled via SMA terminal to a 200 μm fiber (ThorLabs) that was integrated into the microscope light path. Pulse patterns were driven via custom D/A optogenetics software written in Matlab. The stimulation pattern that was used during most *in vivo* calcium imaging experiments consisted of a single 10-ms-long pulse delivered 200 or 100 ms after stimulus presentation every other trial. The light intensity was adjusted to elicit essentially a single spike in most inhibitory neurons, and a moderate and reliable suppression of pyramidal neuron activity without completely suppressing the network. This moderate perturbation had to be adjusted to the cell type. In particular, SOM+ neurons required stronger light intensity to produce a significant suppression of pyramidal cell responses. The absolute values for individual pulses as measured leaving the objective were 15 μW for PV+ and 35 μW for SOM+ interneurons. These values were established in early pilot experiments and were based on previous published work^14^. The effect of ChR2-mediated stimulation was consistent from one experiment to the next so we could keep the same parameters without recalibrating the laser for each experiment (see Supplementary Fig. 7). Note that the effect was systematically assessed after the experiment by examining the average response suppression. To probe PV+ and SOM+ effects, we used single pulse activation of these cells via ChR2 at variable time points relative to the onset of visual stimulation.

### Visual stimulation protocols

Visual stimuli were presented on a 23″ 1080p LCD monitor (Dell) using the software Psychtoolbox-3 on a Windows 7 computer (Dell Precision) with a GeForce 8800 GTS 640 MB graphic card (PNY). The sparse noise stimulus ensemble consisted of black or white squares (150 pixels corresponding to 7.5° in the mouse visual field) displayed at full contrast on a grey background. Each square was presented sequentially in a random location (7 by 12 grid) on the screen for 200 ms followed by a 300 ms duration of background grey before the next stimulation (comprising 2 Hz sparse noise). The location of each square followed a pseudo-random sequence ensuring that two consecutive squares were at least three nodes away from each other to avoid spurious second-order interactions. The polarity of the square (ON or OFF) was random for each square. For experiments including optogenetic stimulation of specific inhibitory neurons, a pulse of blue light was presented every two frames. For the full-field flashes stimulus ensemble, a screen of uniform intensity was displayed for 200 ms followed by a 800 ms duration of background grey (1 Hz). The screen luminance was randomly drawn from 31 values around the background grey ranging from 0 to 100% positive or negative contrast with a maximum luminance of 250 cd m^−2^. Drifting gratings were displayed with a temporal frequency of 2 Hz and a spatial frequency of 0.04 cycles per degree for 36 evenly spaced directions. For each direction, a second of blank screen was followed by 3 s of visual stimulation. Each visual protocol lasted for 40–60 min for calcium imaging and 20–60 min for targeted cell-attached recording.

### Data processing

Spike timing was extracted from the voltage traces using a simple threshold and examining the waveform distribution to ensure that only one cell was recorded. The firing rate over time was estimated by binning each spike train with a 20 ms time bin, matching the calcium imaging sampling frequency for the sake of comparability.

The local baseline average calcium signal was first subtracted from the raw calcium traces acquired at 50 Hz sampling frequency as previously described^14^ to obtain the relative signal *df*/*f*. The resulting signal was high-pass filtered to avoid very slow drift and further deconvolved using a fast non-parametrical algorithm^36^ to extract a signal proportional to the neuron’s actual firing rate at the sampling frequency. A threshold was then applied to the estimated rate to remove part of the noise. For experiments where 10-ms-long pulses of blue light were used to activate specific inhibitory neurons, the calcium signal was saturated during the stimulation time. This artefact was removed from the traces and a linear interpolation was used to reconstruct the calcium signal during the stimulation time bin. Because of the slow calcium time constant and the high-sampling frequency, this did not degrade the signal quality.

### Data analysis

The estimated relative rate as well as the firing rate estimated from cell-attached recordings was used to compute neuronal functional properties in response to sparse noise stimuli or full-field flashes. By averaging these signals following the stimulus onset for each stimulus location and polarity (sparse noise) or luminance (full-field flashes), the spatio-temporal receptive field or the luminance response curve of each recorded neuron could be reconstructed.

To compute the baseline response distribution, we imaged the spontaneous activity of neurons during a 2-min blank screen. We also included imaging data corresponding to stimuli that did not elicit any significant responses (that is, stimuli that were far from receptive fields). We computed the *Z*-score by subtracting the mean value of the baseline distribution from evoked responses and dividing the result by the s.d. of the baseline distribution.

The time course of ON or OFF responses was obtained by averaging spatio-temporal receptive fields over all possible stimulus locations and was further smoothened. A similar baseline distribution was obtained to compute a *Z*-score over time. A neuron sensory response was considered significant if the temporal profile of its *Z*-score crosses the threshold of 3. Response onset was defined as the time point where the response crossed the significance threshold (Z-score >3). The spatial receptive field was obtained by averaging the spatio-temporal receptive fields over times where the response was significantly higher than the baseline activity (Z-score >3). The result was then interpolated and filtered with a Gaussian kernel. Here again, a baseline distribution was used to obtain *Z*-score values. For experiments where comparisons were made between a control condition and activation of specific inhibitory neurons, a time window following the blue light pulse was used where the response was significant compared with baseline and significantly suppressed compared with control. The suppression slope was obtained by fitting a line to the comparison plot. Suppression strength was computed by examining the normalized difference of values between the two conditions relative to the maximum control value.

### Statistical tests

To assess the significance of our results we used paired Wilcoxon signed-rank tests when the same neuron populations were compared between two conditions (in presence or absence of ChR2 stimulation), unpaired *t*-tests when populations of different sizes were compared and ANOVA when more than two unpaired distributions were compared. All tests used in this paper were two-sided. No blinding or randomization of samples was done in any of our analyses. For each experiment and figure, enough data were collected to guarantee the validity of our statistical test. This was generally reflected by the unimodality of population distributions with low s.d. We also performed the normality test and our samples could be described by a Gaussian distribution whenever we used a *t*-test to assess the significance of our results. Variances were computed for all groups and were generally in the same order of magnitude. When this was not the case, as for instance when comparing PV+ and SOM+ response properties, any statistical test resulted in highly significant differences.

### Network model

The network model was similar in overall structure to one recently described^46^. It consisted of 10,000 neurons randomly distributed in a two-dimensional layer. A total of 8,000 of these neurons were excitatory neurons, 1,000 neurons were PV+ neurons and the remaining 1,000 were SOM+ neurons.

All neurons were modelled as conductance-based leaky integrate-and-fire neurons with a membrane time constant τ_m_=25 ms, a leak conductance of *G*_leak_=10 nS and a resting membrane potential of *V*_rest_=−70 mV. The neuron emitted a spike whenever the membrane potential crossed the threshold *V*_th_=−40 mV after which the voltage was artificially clamped at a reset membrane value *V*_reset_=−65 mV for a refractory period of τ_ref_=5 ms. All the values used in the model were in the physiological range^53^,^54^.

Synaptic interactions were modelled as instantaneous increases in excitatory (pyramidal cells) or inhibitory (PV+ and SOM+ neuron) conductances followed by exponential decays with time constants τ_exc_=6 ms and τ_inh_=20 ms. The reversal potentials of the corresponding receptors were *E*_exc_=−0 mV and *E*_inh_=−75 mV. The synaptic weights were *w*_exc_=1.5 nS for excitatory synapses and *w*_inh_=30 nS for inhibitory synapses. The equations for the network dynamics were:




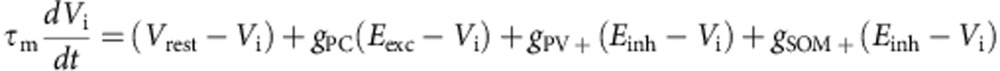







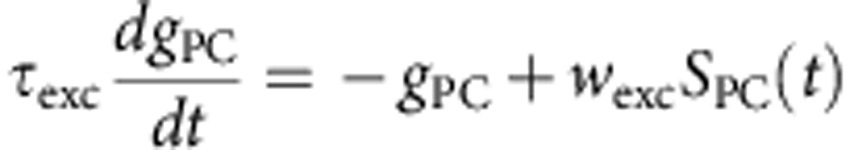







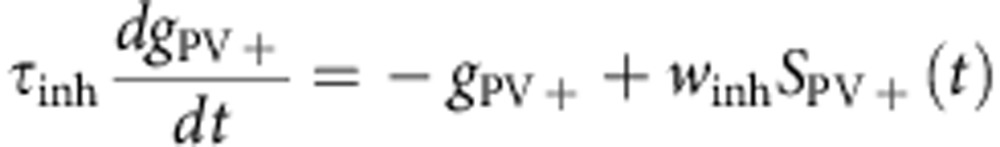







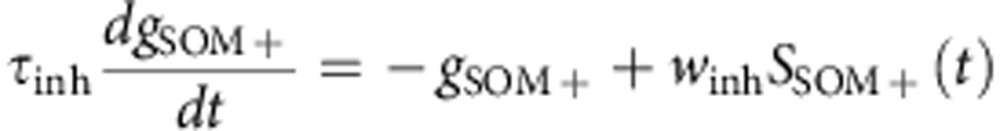




where *i*ε[1−10,000], *g*_PC_, *g*_PV*+*_ and *g*_SOM+_ are expressed in unit of leak conductances and *S*_PC_, *S*_PV*+*_ and *S*_SOM*+*_ are synaptic inputs from pre-synaptic PC, PV+ and SOM+ cells impinging on the post-synaptic neuron *i*.

All neurons in the network had connections with 2% of the rest of the network that were drawn randomly from a distance-dependent distribution. Pyramidal cells and PV+ neurons received inputs and formed projections following a normal distribution with a s.d. of 0.1 mm compared with the network length of 2 mm, whereas SOM+ neurons received inputs from pyramidal cells exclusively and uniformly from the network with four times as many connections compared with other cell types. They also projected their connections to pyramidal cells and PV+ cells with a distance-dependent connectivity rule described by a normal distribution with a s.d. of 0.1 mm. Periodic boundary conditions were assumed for all connections. Interaction delays were also distance dependent with a velocity of 0.1 mm ms^−1^.

To maintain an ongoing regime, all neurons were fed with Poisson processes with an average firing rate of 40 spikes per second and synaptic weights 
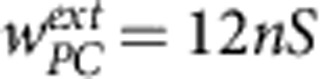
, 
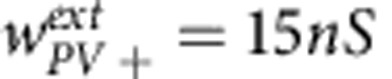
 and 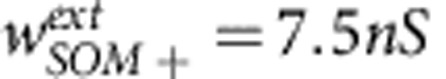. As SOM+ cells did not receive any inhibitory inputs, an additional Poisson input was added to these cells with an average firing rate of 40 spikes per second and synaptic weight of 1.5 nS.

To mimic the sensory input, a two-dimensional external layer projected onto the pyramidal and PV+ neurons of the recurrent network with a 2% density and following a distance-dependent Gaussian distribution with a s.d. of 0.2 mm. To simulate the sparse noise condition, a small Gaussian pattern with spatial s.d. of 0.2 mm, duration of 50 ms and peak average firing rate of 2,000 spikes per second was applied in the external layer. Full-field flashes were simulated by increasing the spatial s.d. to infinity (uniform distribution) and changing the external drive from 0 to 1,000 spikes per second to mimic different luminances. The sensory stimulation was repeated every 500 ms. To mimic channelrhodopsin activation of various inhibitory neuron types, a fixed conductance was injected to all neurons simultaneously at the onset of the sensory response. Values used for PV+ neurons were 20 or 30 nS, and for SOM+ neurons 40 or 50 nS.

All simulations were performed using the NEST simulator^55^ and the PyNN interface^56^.

## Additional information

**How to cite this article**: El-Boustani, S. and Sur, M. Response-dependent dynamics of cell-specific inhibition in cortical networks *in vivo*. *Nat. Commun.* 5:5689 doi: 10.1038/ncomms6689 (2014).

## Supplementary Material

Supplementary Information Supplementary Figures 1-14 and Supplementary References

## References

[b1] Silver R. A. Neuronal arithmetic. Nat. Rev. Neurosci. 11, 474–489 (2010). 20531421 10.1038/nrn2864 PMC4750293

[b2] Haider B. & McCormick D. A. Rapid neocortical dynamics: cellular and network mechanisms. Neuron 62, 171–189 (2009). 19409263 10.1016/j.neuron.2009.04.008 PMC3132648

[b3] Carandini M. & Heeger D. Normalization as a canonical neural computation. Nat. Rev. Neurosci. 13, 51–62 (2012). 10.1038/nrn3136 PMC3273486 22108672

[b4] Katzner S., Busse L. & Carandini M. GABAA inhibition controls response gain in visual cortex. J. Neurosci. 31, 5931–5941 (2011). 21508218 10.1523/JNEUROSCI.5753-10.2011 PMC3083851

[b5] Salinas E. & Thier P. Gain modulation: a major computational principle of the central nervous system. Neuron 27, 15–21 (2000). 10939327 10.1016/s0896-6273(00)00004-0

[b6] McAdams C. J. & Maunsell J. H. Effects of attention on orientation-tuning functions of single neurons in macaque cortical area V4. J. Neurosci. 19, 431–441 (1999). 9870971 10.1523/JNEUROSCI.19-01-00431.1999 PMC6782389

[b7] Treue S. & Martínez Trujillo J. C. Feature-based attention influences motion processing gain in macaque visual cortex. Nature 399, 575–579 (1999). 10376597 10.1038/21176

[b8] Reynolds J. H. & Heeger D. J. The normalization model of attention. Neuron 61, 168–185 (2009). 19186161 10.1016/j.neuron.2009.01.002 PMC2752446

[b9] Somers D., Nelson S. & Sur M. An emergent model of orientation selectivity in cat visual cortical simple cells. J. Neurosci. 75, 5448–5465 (1995). 10.1523/JNEUROSCI.15-08-05448.1995 PMC6577625 7643194

[b10] Anderson J. S. The contribution of noise to contrast invariance of orientation tuning in cat visual cortex. Science 290, 1968–1972 (2000). 11110664 10.1126/science.290.5498.1968

[b11] Ohshiro T., Angelaki D. E. & DeAngelis G. C. A normalization model of multisensory integration. Nat. Neurosci. 14, 775–782 (2011). 21552274 10.1038/nn.2815 PMC3102778

[b12] Louie K. & Glimcher P. W. Separating value from choice: delay discounting activity in the lateral intraparietal area. J. Neurosci. 30, 5498–5507 (2010). 20410103 10.1523/JNEUROSCI.5742-09.2010 PMC2898568

[b13] Ben-Yishai R., Bar-Or R. L. & Sompolinsky H. Theory of orientation tuning in visual cortex. Proc. Natl Acad. Sci. USA 92, 3844–3848 (1995). 7731993 10.1073/pnas.92.9.3844 PMC42058

[b14] Wilson N. R., Runyan C. a., Wang F. L. & Sur M. Division and subtraction by distinct cortical inhibitory networks in vivo. Nature 488, 1–6 (2012). 10.1038/nature11347 PMC3653570 22878717

[b15] Lee S.-H. . Activation of specific interneurons improves V1 feature selectivity and visual perception. Nature 488, 379–383 (2012). 22878719 10.1038/nature11312 PMC3422431

[b16] Doiron B., Longtin A., Berman N. & Maler L. Subtractive and divisive inhibition: effect of voltage-dependent inhibitory conductances and noise. Neural Comput. 13, 227–248 (2001). 11177434 10.1162/089976601300014691

[b17] Holt G. R. & Koch C. Shunting inhibition does not have a divisive effect on firing rates. Neural Comput. 9, 1001–1013 (1997). 9188191 10.1162/neco.1997.9.5.1001

[b18] Mitchell S. J. & Silver R. A. Shunting inhibition modulates neuronal gain during synaptic excitation. Neuron 38, 433–445 (2003). 12741990 10.1016/s0896-6273(03)00200-9

[b19] Murphy B. K. & Miller K. D. Multiplicative gain changes are induced by excitation or inhibition alone. J. Neurosci. 23, 10040–10051 (2003). 14602818 10.1523/JNEUROSCI.23-31-10040.2003 PMC6740852

[b20] Ascoli G. a. . Petilla terminology: nomenclature of features of GABAergic interneurons of the cerebral cortex. Nat. Rev. Neurosci. 9, 557–568 (2008). 18568015 10.1038/nrn2402 PMC2868386

[b21] Markram H. . Interneurons of the neocortical inhibitory system. Nat. Rev. Neurosci. 5, 793–807 (2004). 15378039 10.1038/nrn1519

[b22] Moore C. I., Carlen M., Knoblich U. & Cardin J. a. Neocortical interneurons: from diversity, strength. Cell 142, 189–193 (2010). 20655460 10.1016/j.cell.2010.07.005 PMC3655709

[b23] Petersen C. C. H. & Crochet S. Synaptic computation and sensory processing in neocortical layer 2/3. Neuron 78, 28–48 (2013). 23583106 10.1016/j.neuron.2013.03.020

[b24] Alitto H. J. & Dan Y. Function of inhibition in visual cortical processing. Curr. Opin. Neurobiol. 20, 340–346 (2010). 20307968 10.1016/j.conb.2010.02.012 PMC3572778

[b25] Pfeffer C. K., Xue M., He M., Huang Z. J. & Scanziani M. Inhibition of inhibition in visual cortex: the logic of connections between molecularly distinct interneurons. Nat. Neurosci. 16, 1068–1076 (2013). 23817549 10.1038/nn.3446 PMC3729586

[b26] Atallah B., Bruns W., Carandini M. & Scanziani M. Parvalbumin-expressing interneurons linearly transform cortical responses to visual stimuli. Neuron 73, 159–170 (2012). 22243754 10.1016/j.neuron.2011.12.013 PMC3743079

[b27] Lee S.-H., Kwan A. C. & Dan Y. Interneuron subtypes and orientation tuning. Nature 508, E1–E2 (2014). 24695313 10.1038/nature13128

[b28] El-Boustani S., Wilson N. R., Runyan C. a. & Sur M. El-Boustani et al. reply. Nature 508, E3–E4 (2014). 10.1038/nature13130 24695315

[b29] Kulik A. . Compartment-dependent colocalization of Kir3.2-containing K+ channels and GABAB receptors in hippocampal pyramidal cells. J. Neurosci. 26, 4289–4297 (2006). 16624949 10.1523/JNEUROSCI.4178-05.2006 PMC6673994

[b30] Nusser Z., Sieghart W., Benke D., Fritschy J. M. & Somogyi P. Differential synaptic localization of two major gamma-aminobutyric acid type A receptor alpha subunits on hippocampal pyramidal cells. Proc. Natl Acad. Sci. USA 93, 11939–11944 (1996). 8876241 10.1073/pnas.93.21.11939 PMC38162

[b31] Chance F. S., Abbott L. F. & Reyes A. D. Gain modulation from background synaptic input. Neuron 35, 773–782 (2002). 12194875 10.1016/s0896-6273(02)00820-6

[b32] Abbott L. F. & Chance F. S. Drivers and modulators from push-pull and balanced synaptic input. Prog. Brain Res. 149, 147–155 (2005). 16226582 10.1016/S0079-6123(05)49011-1

[b33] Ma W. . Visual representations by cortical somatostatin inhibitory neurons-selective but with weak and delayed responses. J. Neurosci. 30, 14371–14379 (2010). 20980594 10.1523/JNEUROSCI.3248-10.2010 PMC3001391

[b34] Adesnik H., Bruns W., Taniguchi H., Huang Z. J. & Scanziani M. A neural circuit for spatial summation in visual cortex. Nature 490, 226–231 (2012). 23060193 10.1038/nature11526 PMC3621107

[b35] Liu B. . Visual receptive field structure of cortical inhibitory neurons revealed by two-photon imaging guided recording. J. Neurosci. 29, 10520–10532 (2009). 19710305 10.1523/JNEUROSCI.1915-09.2009 PMC2779138

[b36] Vogelstein J. T. . Fast nonnegative deconvolution for spike train inference from population calcium imaging. J. Neurophysiol. 104, 3691–3704 (2010). 20554834 10.1152/jn.01073.2009 PMC3007657

[b37] Yeh C.-I., Xing D., Williams P. E. & Shapley R. M. Stimulus ensemble and cortical layer determine V1 spatial receptive fields. Proc. Natl Acad. Sci. USA 106, 14652–14657 (2009). 19706551 10.1073/pnas.0907406106 PMC2732842

[b38] Bonin V., Histed M. H., Yurgenson S. & Reid R. C. Local diversity and fine-scale organization of receptive fields in mouse visual cortex. J. Neurosci. 31, 18506–18521 (2011). 22171051 10.1523/JNEUROSCI.2974-11.2011 PMC3758577

[b39] Xu H., Jeong H.-Y., Tremblay R. & Rudy B. Neocortical somatostatin-expressing GABAergic interneurons disinhibit the thalamorecipient layer 4. Neuron 77, 155–167 (2013). 23312523 10.1016/j.neuron.2012.11.004 PMC3556168

[b40] Cottam J. C. H., Smith S. L. & Häusser M. Target-specific effects of somatostatin-expressing interneurons on neocortical visual processing. J. Neurosci. 33, 19567–19578 (2013). 24336721 10.1523/JNEUROSCI.2624-13.2013 PMC3858626

[b41] Cardin J. a. . Driving fast-spiking cells induces gamma rhythm and controls sensory responses. Nature 459, 663–667 (2009). 19396156 10.1038/nature08002 PMC3655711

[b42] Haider B., Häusser M. & Carandini M. Inhibition dominates sensory responses in the awake cortex. Nature 493, 97–100 (2013). 23172139 10.1038/nature11665 PMC3537822

[b43] Polack P.-O., Friedman J. & Golshani P. Cellular mechanisms of brain state-dependent gain modulation in visual cortex. Nat. Neurosci. 16, 1331–1339 (2013). 23872595 10.1038/nn.3464 PMC3786578

[b44] Pi H.-J. . Cortical interneurons that specialize in disinhibitory control. Nature 503, 521–524 (2013). 24097352 10.1038/nature12676 PMC4017628

[b45] Fu Y. . A cortical circuit for gain control by behavioral state. Cell 156, 1139–1152 (2014). 24630718 10.1016/j.cell.2014.01.050 PMC4041382

[b46] Yger P., El Boustani S., Destexhe A. & Frégnac Y. Topologically invariant macroscopic statistics in balanced networks of conductance-based integrate-and-fire neurons. J. Comput. Neurosci. 31, 229–245 (2011). 21222148 10.1007/s10827-010-0310-z

[b47] Softky W. R. & Koch C. The highly irregular firing of cortical cells is inconsistent with temporal integration of random EPSPs. J. Neurosci. 13, 334–350 (1993). 8423479 10.1523/JNEUROSCI.13-01-00334.1993 PMC6576320

[b48] Herman A. M., Huang L., Murphey D. K., Garcia I. & Arenkiel B. R. Cell type-specific and time-dependent light exposure contribute to silencing in neurons expressing Channelrhodopsin-2. Elife 3, e01481–e01481 (2014). 24473077 10.7554/eLife.01481 PMC3904216

[b49] Atallah B. V., Scanziani M. & Carandini M. Atallah et al. reply. Nature 508, E3 (2014). 10.1038/nature13129 24695314

[b50] Arroyo S., Bennett C., Aziz D., Brown S. P. & Hestrin S. Prolonged disynaptic inhibition in the cortex mediated by slow, non-α7 nicotinic excitation of a specific subset of cortical interneurons. J. Neurosci. 32, 3859–3864 (2012). 22423106 10.1523/JNEUROSCI.0115-12.2012 PMC3320796

[b51] Gentet L. J. . Unique functional properties of somatostatin-expressing GABAergic neurons in mouse barrel cortex. Nat. Neurosci. 15, 1–7 (2012). 10.1038/nn.3051 22366760

[b52] Chen T.-W. . Ultrasensitive fluorescent proteins for imaging neuronal activity. Nature 499, 295–300 (2013). 23868258 10.1038/nature12354 PMC3777791

[b53] Vogels T. P. & Abbott L. F. Signal propagation and logic gating in networks of integrate-and-fire neurons. J. Neurosci. 25, 10786–10795 (2005). 16291952 10.1523/JNEUROSCI.3508-05.2005 PMC6725859

[b54] Kumar A., Schrader S., Aertsen A. & Rotter S. The high-conductance state of cortical networks. Neural Comput. 20, 1–43 (2008). 18044999 10.1162/neco.2008.20.1.1

[b55] Diesmann M. & Gewaltig M.-O. inForschung und wisschenschaftliches Rechnen, Beiträge zum Heinz-Billing-Preis 2001 Vol. 58 (eds Plesser, T. & Macho, V.)43–70 (2002).

[b56] Davison A. P. . PyNN: a common interface for neuronal network simulators. Front. Neuroinform. 2, 11 (2008). 19194529 10.3389/neuro.11.011.2008 PMC2634533

