# Supplementary material for: Response-dependent dynamics of cell-specific inhibition in cortical networks in vivo
Source: Nat Commun. 2014 Dec 11;5:5689. doi: 10.1038/ncomms6689 (PMC4268659; doi:10.1038/ncomms6689)
Supplement: Supplementary Information — Supplementary Figures 1-14 and Supplementary References [file ncomms6689-s1.pdf]

SUPPLEMENTARY INFORMATION

SUPPLEMENTARY FIGURES

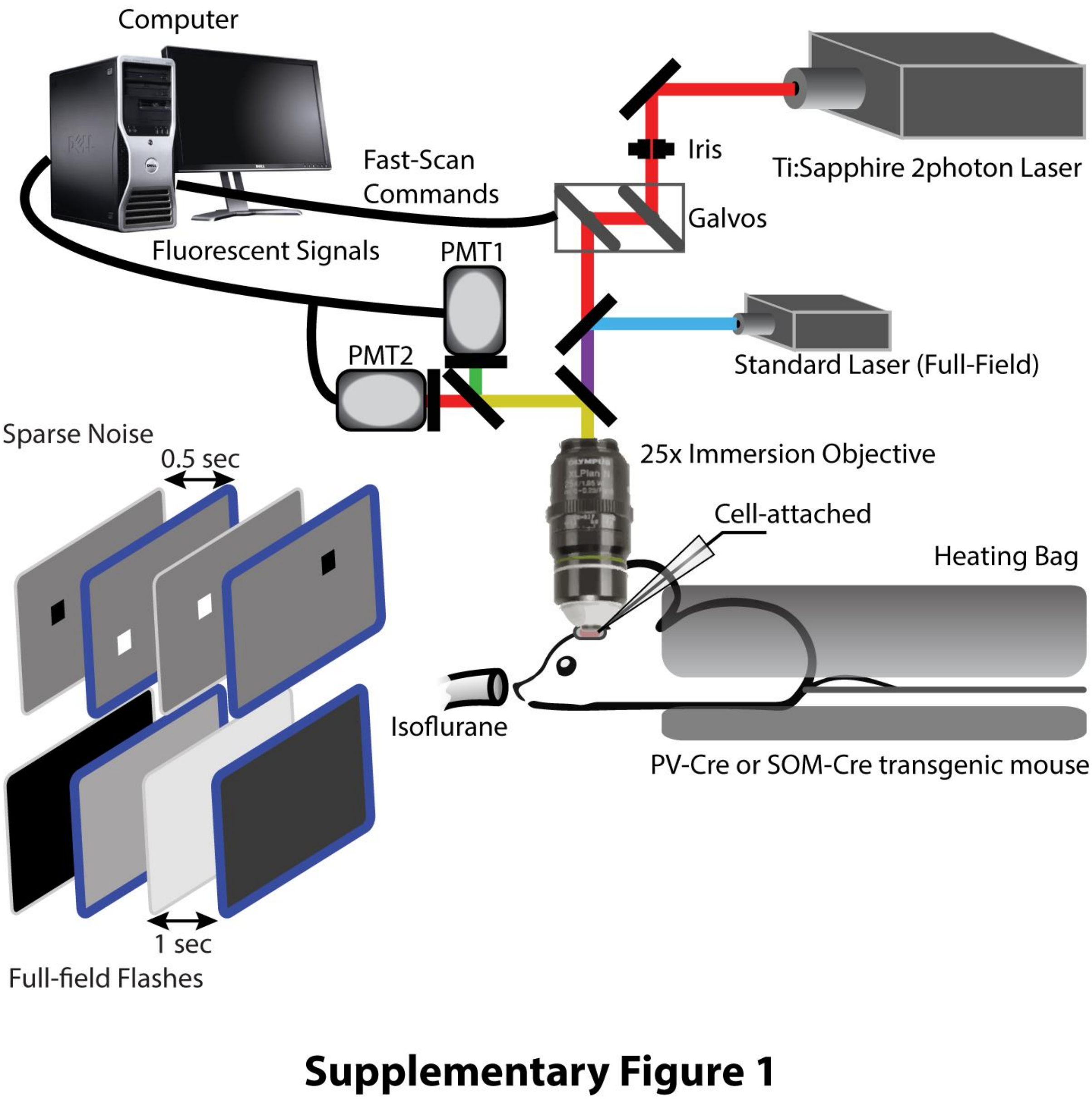

Supplementary Figure 1. Schematic of the experimental design described in the Methods section.

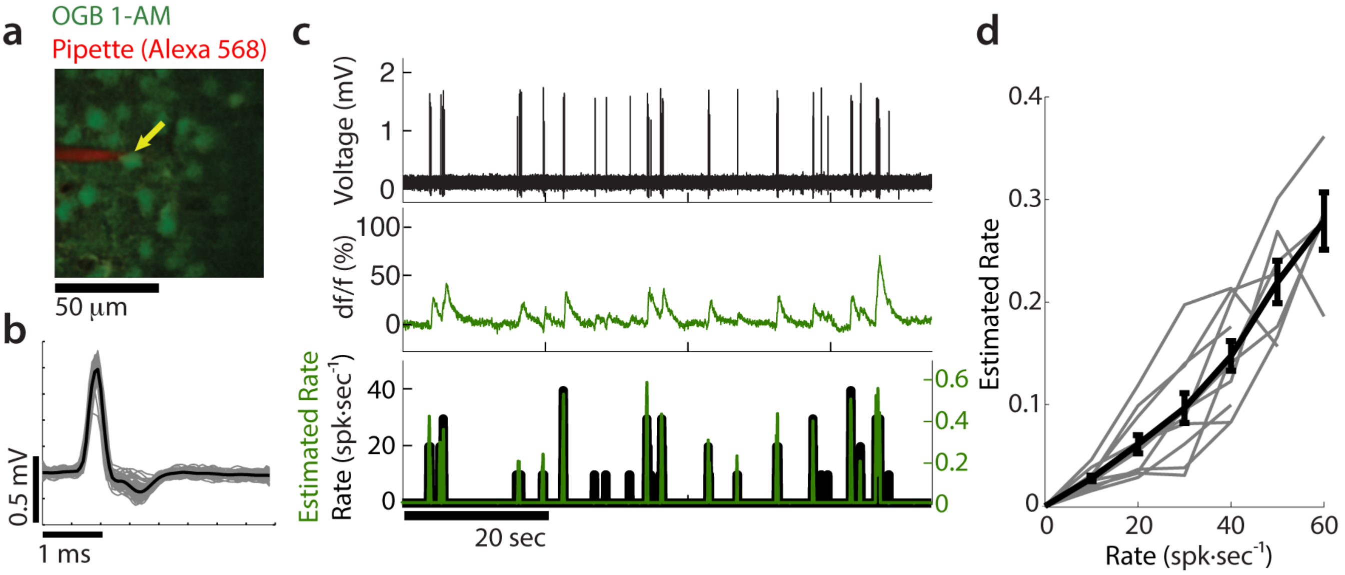

Supplementary Figure 2

Supplementary Figure 2. Deconvolution of spike rate from calcium traces. **(a)** *In-vivo* targeted cell-attached recording of a neuron loaded with OGB 1-AM (yellow arrow) using a glass pipette filled with Alexa 568. **(b)** Spike waveforms of the targeted neuron. The average waveform is shown in black line. **(c)** Simultaneous recording and imaging of the neuron targeted in **a**. Top: Voltage trace obtained by cell-attached recording. Middle: Calcium response obtained with high-speed calcium imaging. Bottom: Spike rate computed from the cell-attached recording (black, time bin=20 ms) and reconstructed spike rate from the calcium trace using a fast deconvolution algorithm[1] (green, time bin: 20ms). Both signals were convolved with a 100ms square function prior to comparison. Pearson correlation coefficient between the two traces: 0.8. The estimated firing rate for all individual traces was well correlated with the measured firing rate sampled at the same frequency (Pearson's coefficient: 0.77±0.04), in accordance with previously reported values[2,3]. **(d)** Estimated spike rate obtained by deconvolution as a function of the number of spikes per bin for n=11 trials (2 minutes long each) for the cell targeted in **a** (gray curves). The average curve is shown in black, with bars representing standard error of the mean (slope=0.047, r=0.9, p<0.001, paired t-test).

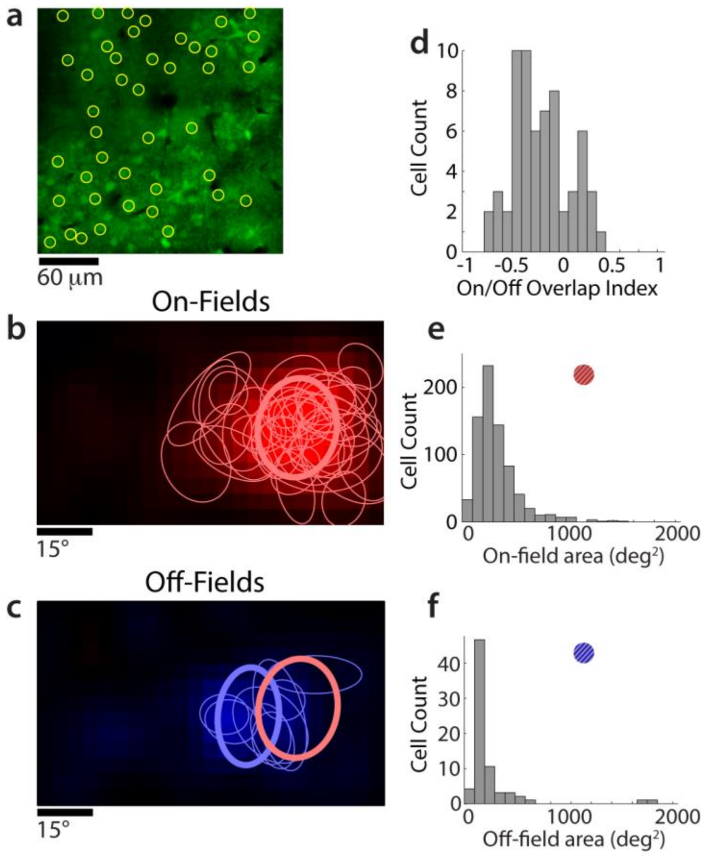

Supplementary Figure 3

Supplementary Figure 3. Properties of ON and OFF responses as measured with sparse noise stimuli during calcium imaging experiments. We first observed that ON or OFF receptive fields of simultaneously recorded neurons were highly overlapping indicating that nearby neurons tend to encode similar sensory information[4]. The area of the receptive fields was estimated by fitting a two-dimensional Gaussian function[5]. **(a)** Baseline image used to identify cells loaded with OGB-1AM. Neurons displaying significant visual responses are marked with yellow circles. **(b)** Distribution of the corresponding neuronal ON (red) and **(c)** OFF (blue) receptive fields represented by ellipses obtained with Gaussian fits. The ellipses delimit the circumferences of the Gaussian fits as well as their orientation. Population average receptive fields are shown in the background and the corresponding Gaussian fits are represented in thick lines. **(d)** Distribution of ON/OFF receptive field overlap index for all neurons in the dataset that displayed significant responses for both stimuli (n=71). **(e)** ON receptive fields areas (n=751, mean = 270 deg^2) and **(f)** OFF receptive fields areas (n=81, mean = 140 deg^2). The size of each receptive field was estimated by computing the area within the ellipse. These values were compatible with those reported previously[2,4,6].

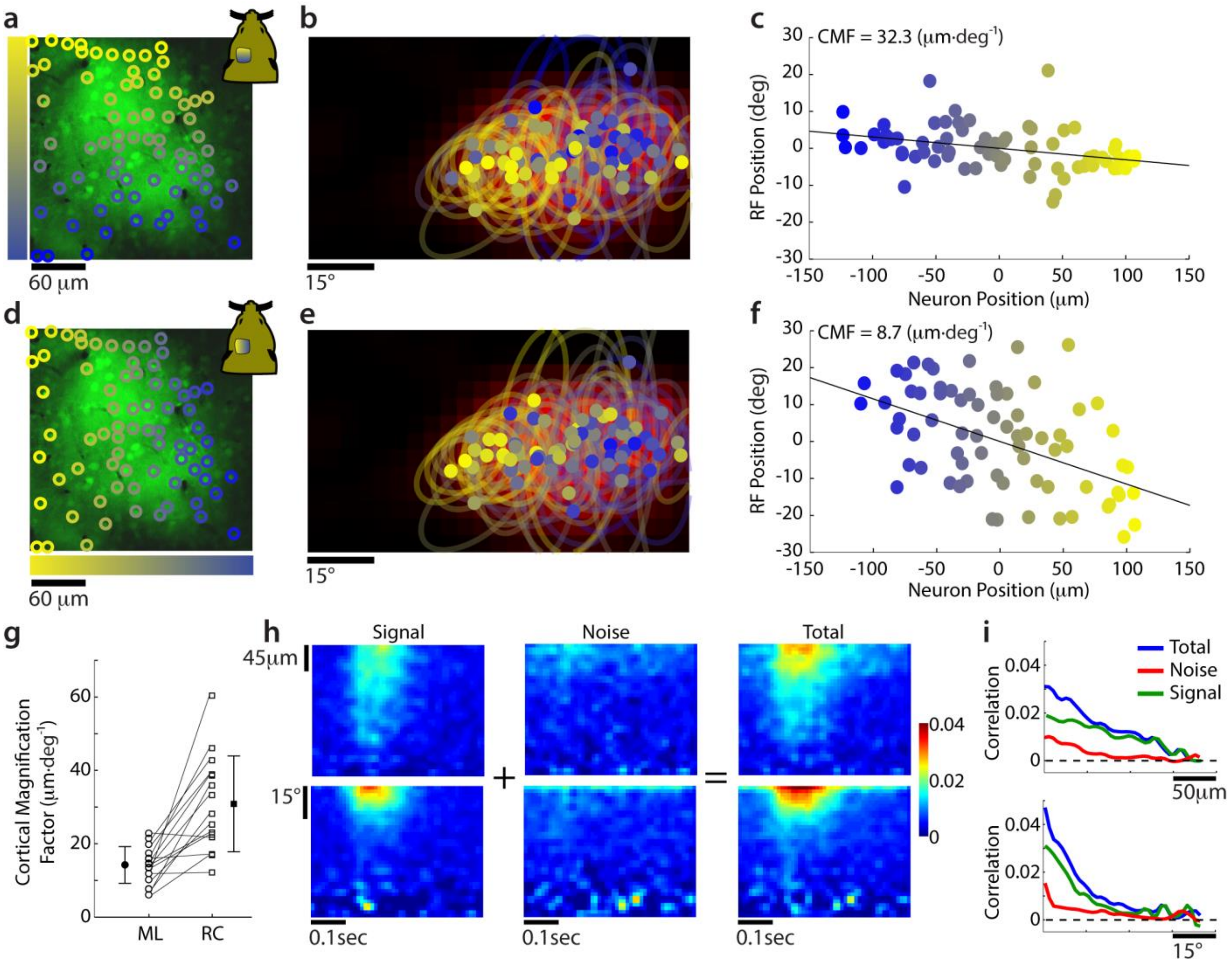

Supplementary Figure 4

Supplementary Figure 4. Functional organization of local neuron populations in V1. Considering ON fields alone, we examined the retinotopic organization of simultaneously imaged neurons. **(a)** Rostrocaudal labeling of individual imaged neurons. The color code represents rostral (yellow) neurons to caudal (blue) neurons, as depicted by the mouse schematic. **(b)** Distribution of individual receptive fields with a color label corresponding to the neuronal location in **a**. Centers of the receptive fields are marked by filled circles and the spatial extent is illustrated with transparent ellipses. The population average receptive field is shown in the background. **(c)** Neuronal receptive field positions in the screen plotted as a function of the location of the corresponding neurons in the cortex. Linear fit is shown with a black line (cortical magnification factor, CMF: 32.3 μm deg^{-1}). **(d-f)** Same as **a-c** for the mediolateral axis (CMF: 8.7 μm deg^{-1}). The neuronal receptive field location on the screen was linearly related to the physical location of these neurons on the cortex in the rostro-caudal and medio-lateral axes, as previously described

with more complex stimulus ensembles[2]. **(g)** Comparison between the mediolateral (ML) and rostrocaudal (RC) axis cortical magnification factor for different experiments (N=15). The cortical magnification factor estimated from these distributions was in line with previously reported values using intrinsic imaging[7]. **(h)** Average pairwise correlation as a function of time relative to the stimulus onset and cortical distance (top) or receptive field distance (bottom). The total correlation (right) is the sum of signal correlation (left) and noise correlation (center). The former matrix could be obtained by shuffling all trials corresponding to the same stimulus for each pair of cells. The noise correlation matrix was obtained as the difference between the total correlation matrix and the signal correlation matrix. **(i)** Average total (blue), noise (red) and signal (green) pairwise correlation as a function of cortical distance (top) or receptive field distance (bottom) averaged over responsive time windows in **h**. Although the noise correlation was weaker and stimulus-independent, it displayed a significant distance-dependent decay indicating that nearby neurons tended to be more correlated than distant neurons even in absence of sensory drive.

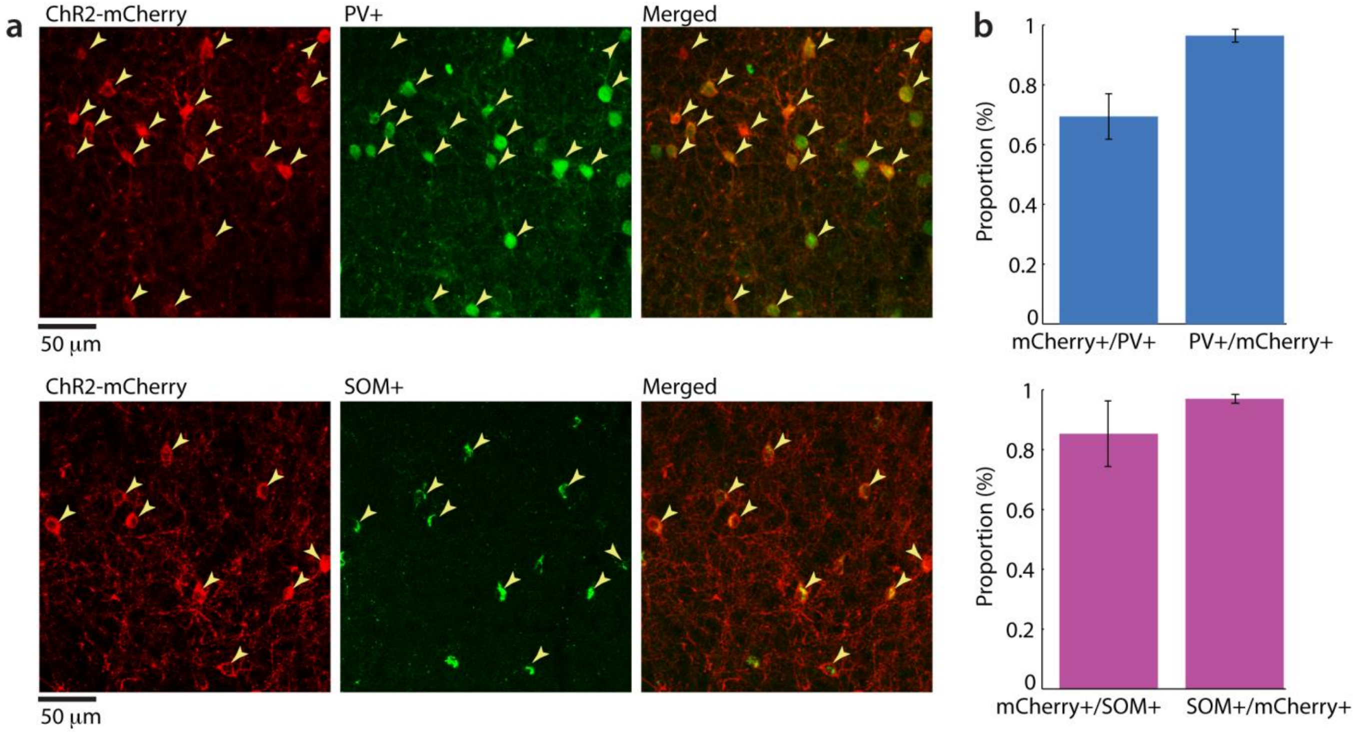

Supplementary Figure 5

Supplementary Figure 5. (a) Immunohistochemical staining of coronal sections from V1 of PV-Cre (top, 159 neurons in n=5 sections, 2 animals) and SOM-Cre (bottom, 101 neurons, n=8 sections, 2 animals) mice injected with Cre-dependent mCherry-ChR2. The left column shows the expression of the injected construct (mCherry) in the red channel. The middle column shows the immunostaining labeling of either PV+ or SOM+ neurons. The right column shows the merged image with co-localization of identified cells. **(b)** Quantification of the expression specificity and coverage for PV-Cre (top) and SOM-Cre (bottom) mice. The left bar indicates in the vicinity of the injection site the proportion of PV+ or SOM+ neurons that expressed mCherry-ChR2. The right bar indicates the proportion of neurons expressing mCherry-ChR2 that successfully expressed in the target population (PV+ or SOM+). Error bars are standard error of the mean. This analysis shows that the expression is highly specific to the targeted population and covers majority of the PV+ or SOM+ interneurons in the injected region.

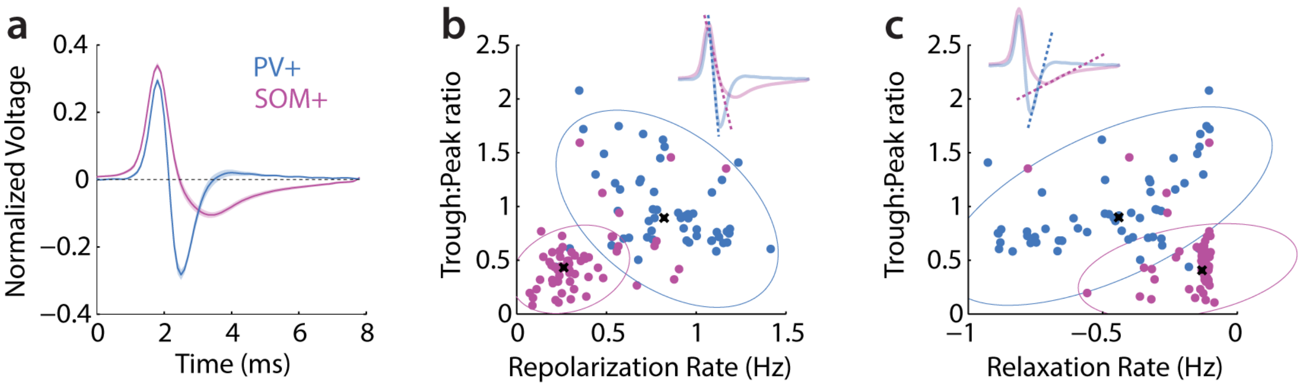

Supplementary Figure 6

Supplementary Figure 6. Post-hoc spike waveform analysis of identified PV+ and SOM+ interneurons recorded with targeted cell-attached. **(a)** Average normalized spike waveform for all the recorded PV+ (blue, n=56) and SOM+ (pink, n=59). Standard errors of the mean are depicted with shaded areas. The average spike waveform for PV+ interneurons is of the fast-spiking type whereas that for SOM+ neurons is of the regular-spiking type. **(b)** Distribution of individual spike waveforms as a function of trough-to-peak ratio and repolarization rate (see inset). **(c)** Distribution of individual spike waveforms as a function of trough-to-peak ratio and relaxation rate (see inset). In **b** and **c** the black crosses indicate the center of each cluster for PV+ and SOM+ neurons and the ellipses indicates the 95% confidence interval as assessed by principal component analysis. Both representations show that the classification done during the experiment by eliciting reliable and precise spikes through ChR2 was in good agreement with the waveform classification: the centroid of each cluster computed for PV+ or SOM+ neurons is not included in the confidence interval of the other cluster that was computed without prior knowledge of the cell-type.

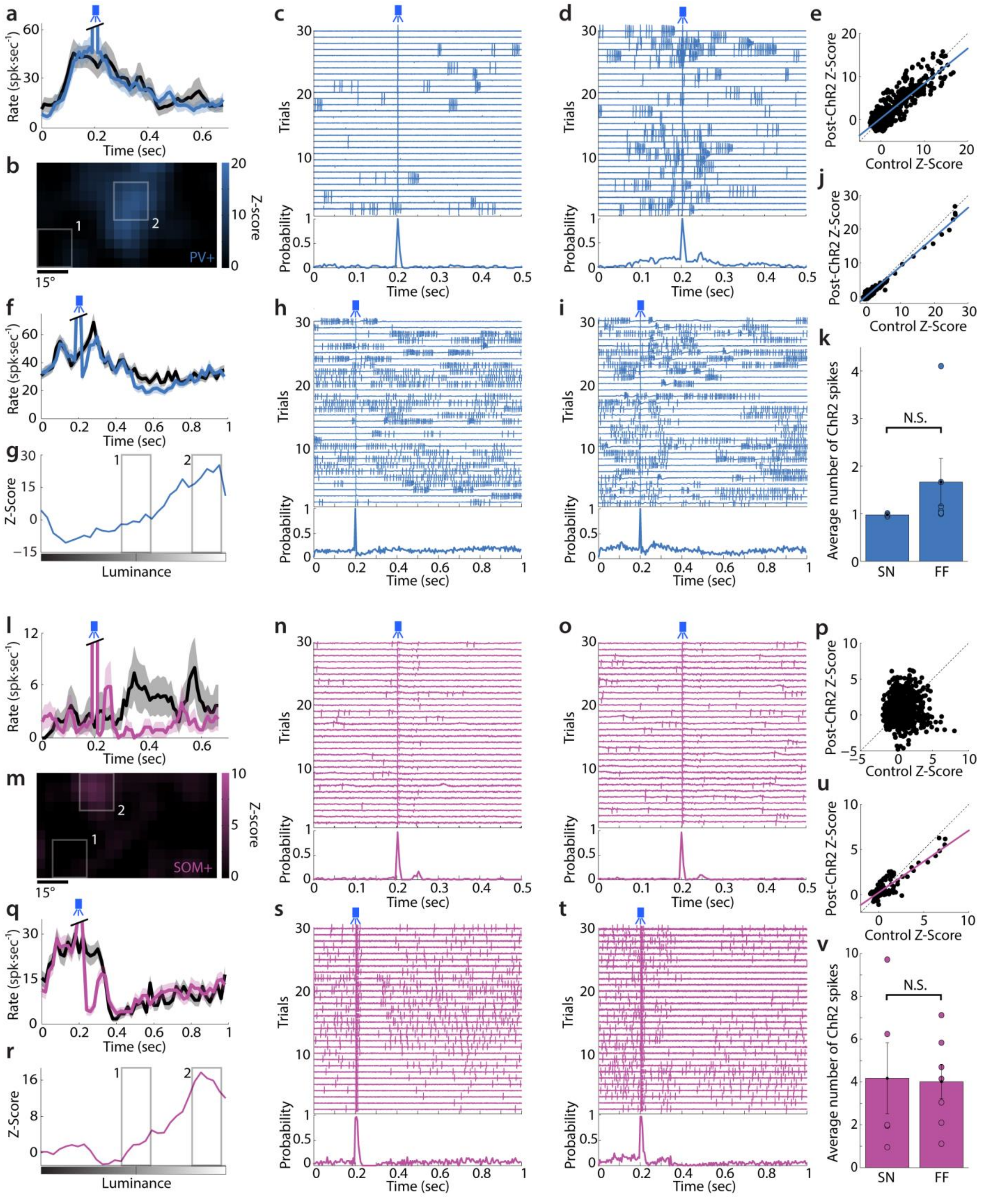

Supplementary Figure 7

Supplementary Figure 7. Precise and reliable control of PV+ and SOM+ neuron firing with ChR2 for varying levels of visually evoked activity. **(a)** Temporal response profile of a PV+ neuron

expressing ChR2-mCherry obtained by *in vivo* targeted cell-attached recording in response to sparse noise stimuli in absence (black) or presence (blue) of a light pulse at 0.2 sec. Standard errors of the mean are indicated by shaded areas. **(b)** ON field of the neuron targeted in **a**. Two regions are highlighted, corresponding respectively to the center of the receptive field (2) and a region well outside the receptive field (1). **(c)** Top: recordings corresponding to trials where stimuli were presented in region 1. Bottom: spiking probability for each time point computed over all trials with a 5ms time bin. **(d)** Same as **c** for the region 2 in **b**. **(e)** Point-by-point comparison of responses (averaged over 100ms following pulse) at all stimulus locations between control condition and with ChR2-activation of PV+ neurons (n=3). Individual scatter plots were centered (linear fit: slope=0.8, r=0.87, $p<0.001$, paired t-test). **(f-j)** Same as **a-e** for PV+ neurons (n=6) expressing ChR2-mCherry during the full-field flash stimulation. **(g)** Two regions are indicated by gray squares corresponding to high visual responses (2) and baseline activity (1). **(j)** Slope=0.87, r=0.99, $p<0.001$, paired t-test. **(k)** Average number of ChR2-elicited spikes during visually evoked activity with sparse noise or full-field flashes (p=0.38, unpaired t-test). Dots show values for single cells. **(l-p)** Same as **a-e** for SOM+ neurons (n=5) expressing ChR2-mCherry during sparse noise stimulation. **(p)** Slope=0.05, r=0.04, p=0.14, paired t-test. **(q-u)** Same as **f-j** for SOM+ neurons (n=7) expressing ChR2-mCherry during the full-field flash stimulation. **(u)** Slope=0.7, r=0.86, $p<0.001$, paired t-test. **(v)** Same as **k** during SOM+ neurons stimulation (p=0.92, unpaired t-test). This analysis indicates that there is little interaction between visually evoked activity and spikes evoked by the blue light pulse. The average number of evoked spikes is independent of the visual stimulus, for both PV+ neurons (average of ~1.7 spikes per pulse, n=9, 3 animals) and SOM+ (average of ~4.1 spikes per pulse, n=12, 3 animals).

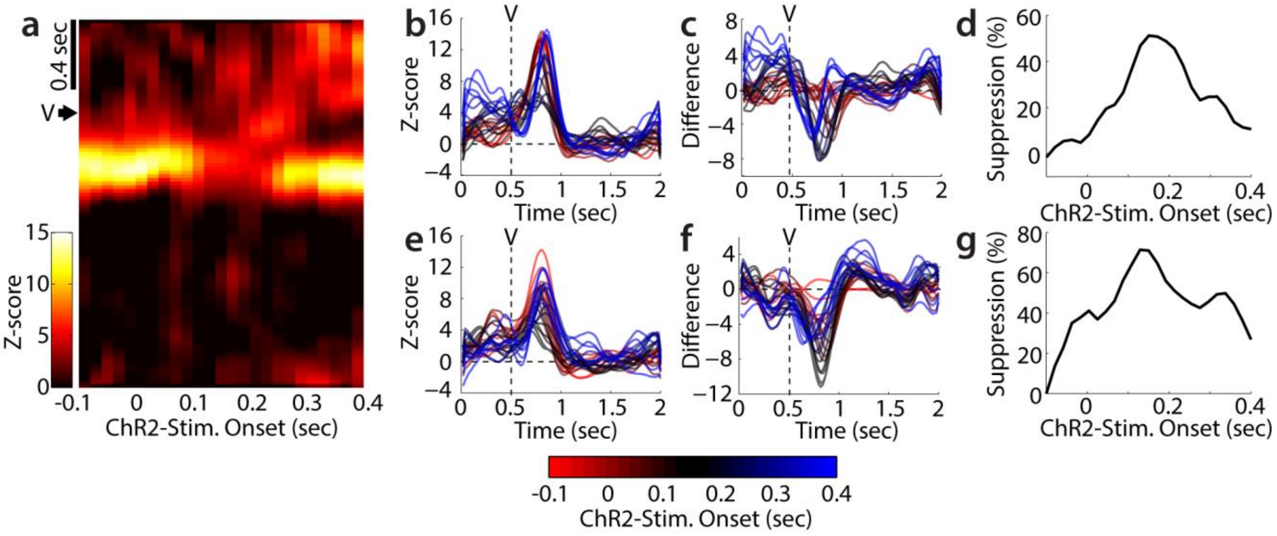

Supplementary Figure 8

Supplementary Figure 8. Study of ChR2 stimulation times relative to visual stimulus onset, and the resulting effect on population responses. Shown are example experiments demonstrating the effect of single pulse SOM+ neuron activation on pyramidal neuron responses to full-field flashes. **(a)** Population activity time course averaged over all simultaneously imaged neurons with significant responses (n=45) for full-field flashes and for various SOM+ cell ChR2 stimulation times relative to the onset of the stimulus. Values range from 100ms prior to the visual stimulus onset to 400ms after visual stimulus onset. The letter 'V' indicates the timing of visual stimulus onset. Visual responses are suppressed most when the ChR2 pulse onset is around 0.2 sec. **(b)** Population activity time course for various ChR2 stimulation times relative to the onset of the stimulus. Early timings are color coded in red and late timings in blue. **(c)** Suppression time course as measured by taking the difference between the control response in absence of ChR2 stimulation and the responses after ChR2 stimulation. Color code is same as panel **b**. **(d)** Suppression amplitude relative to the control response as a function of ChR2 stimulation time. The suppression amplitude was computed by averaging curves in **c** normalized to the control response. **(e-g)** Same as **b-d** for another experiment.

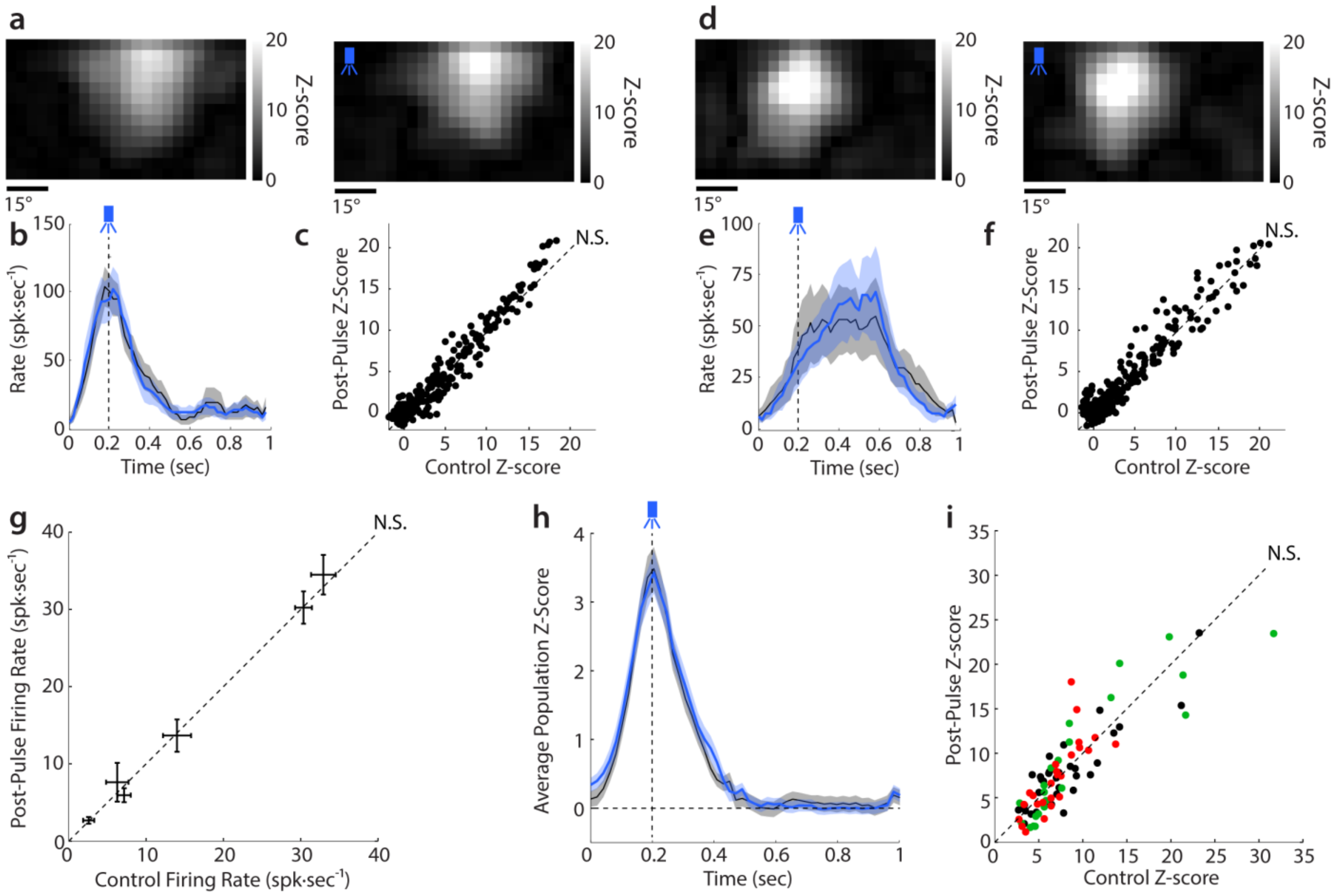

Supplementary Figure 9

Supplementary Figure 9. Blue light pulses do not affect neuron responses or the visual system in absence of ChR2. **(a-g)** Cell-attached recordings from unidentified neurons were performed in wild-type mice during presentation of sparse noise stimuli and blue light pulses. **(a)** The spatial ON field in the control condition (left) and in presence of blue light stimulation (right). **(b)** Temporal profile of the evoked activity averaged over all stimuli presentation and binned with a 20ms bin size. The control condition is shown in black and the condition with blue light in blue. Standard errors of the mean are shown as shaded areas. The timing of the blue light pulse is indicated with a vertical dashed line. **(c)** Point-by-point comparison of the spatial ON field between both conditions (N.S. Not Significant, p=0.84, Wilcoxon signed-rank test). **(d-f)** Same as **a-c** for another recorded cell (N.S. Not Significant, p=0.31, Wilcoxon signed-rank test). **(g)** Comparison between average responses measured in the control condition and in presence of blue light pulses for all the recorded cells (n=6, 1 animal, N.S. Not Significant, p=0.75, Wilcoxon signed-rank test). Error bars indicate standard error of the mean. **(h-i)** The same experiment was performed with calcium imaging to verify that our result is not an artifact due to interference of

blue light pulses with the imaging. **(h)** Population z-score obtained by averaging the responses over all stimuli and all the simultaneously imaged cells with significant responses (n=31). Standard errors of the mean are shown as shaded areas. The timing of the blue light pulse is indicated with a vertical dashed line. **(i)** Results of three different experiments (shown in different colors), comparing the average z-score of individual target cells in the control condition and with blue light stimulation (n=82, p=0.45, Wilcoxon signed-rank test). These results indicate that the effects observed in this study were all mediated by ChR2 excitation of specific interneurons and were not the result of spurious data or uncontrolled effect on the visual system. Note that all these controls were made with the laser intensity set at 40μW which is the largest value used in this study.

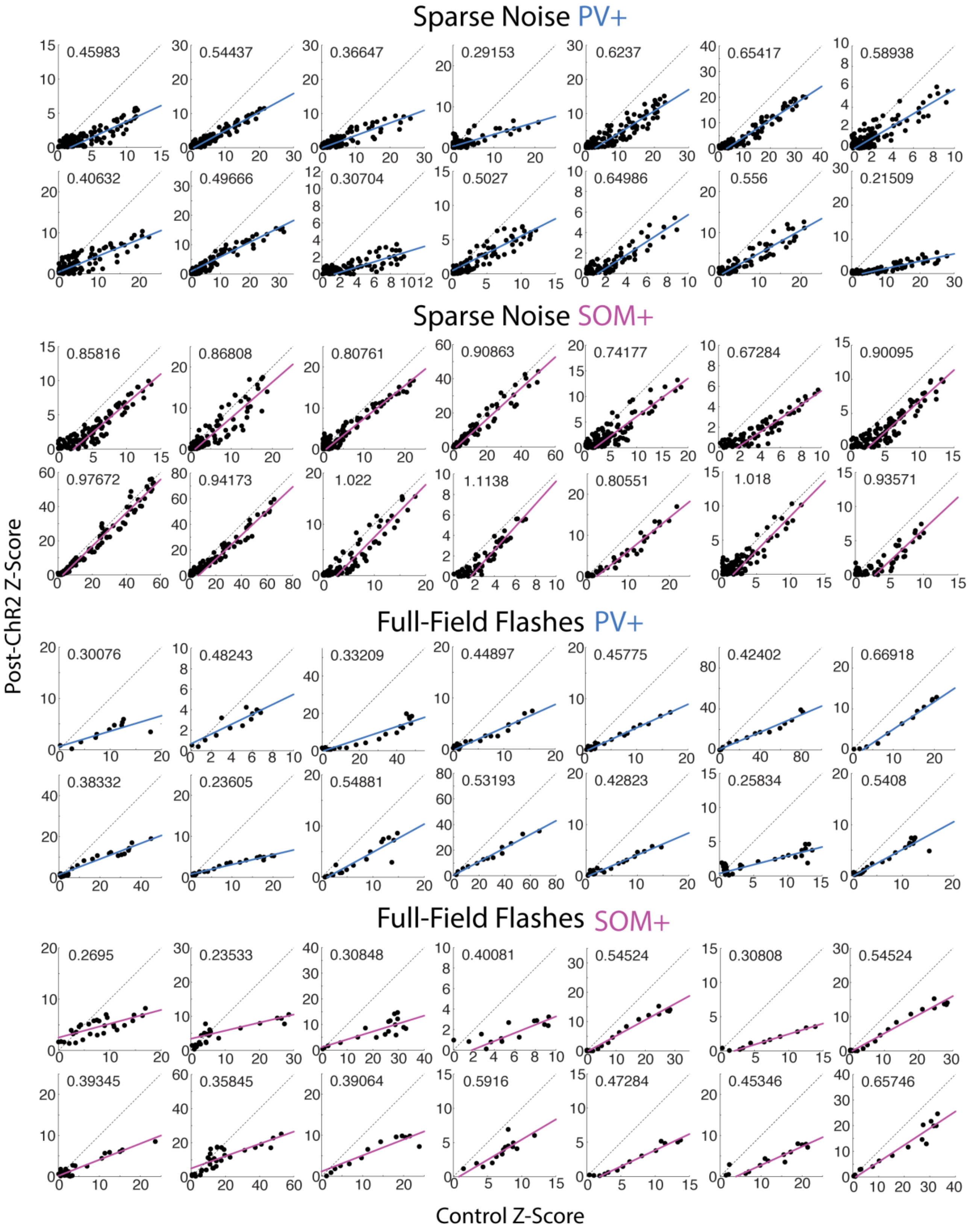

Supplementary Figure 10

Supplementary Figure 10. Examples of the effect of ChR2-mediated PV+ or SOM+ neuron activation on individual target cells during sparse noise (top) or full-field flash (bottom) stimuli. In each panel, the slope of the fitted line is indicated in the upper left corner.

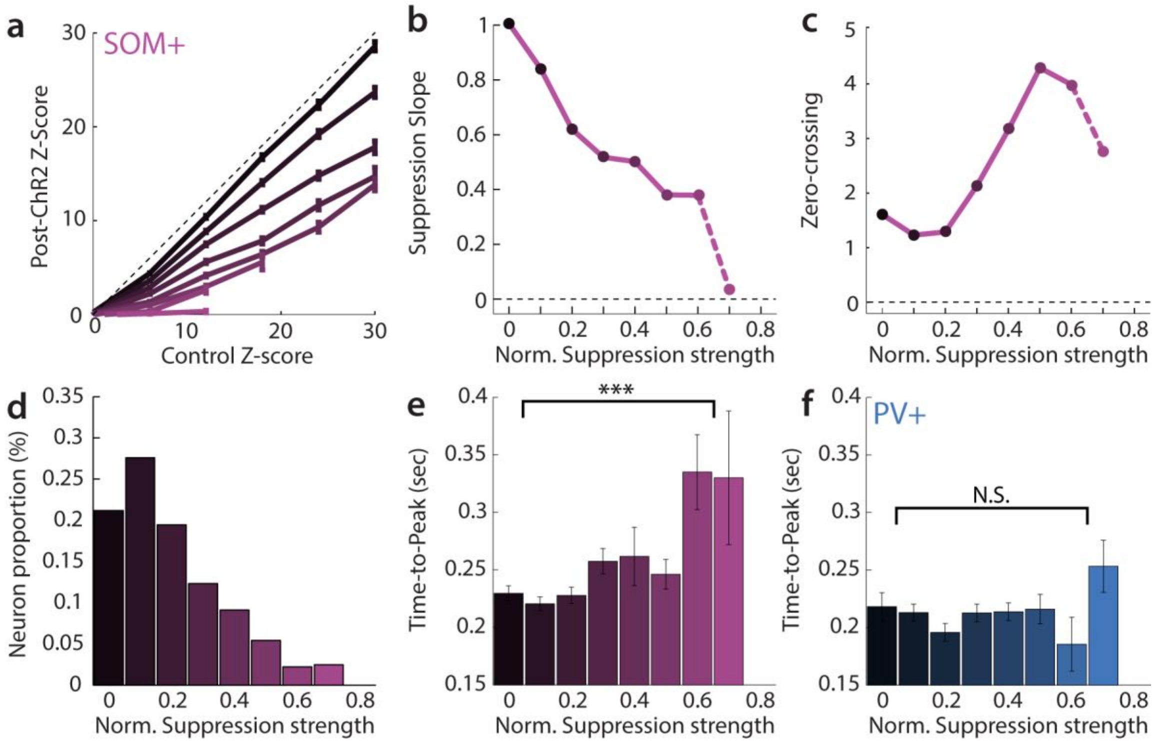

Supplementary Figure 11

Supplementary Figure 11. Analysis of pyramidal cell response suppression by SOM+ neurons during sparse noise stimulation. We analyzed groups of pyramidal cells that displayed various levels of suppression strength (as quantified by the normalized difference between the total evoked activity observed during control and ChR2-activation conditions). **(a)** Comparison of the population z-score averaged over all neurons displaying similar suppression strength, between the control condition and when SOM+ neurons were stimulated (n=406). Different curves are color coded according to the normalized suppression strength ranging from 0 (black) to 0.7 (pink). **(b-c)** Parameters of the threshold-linear fit for the curves in **a.** **(b)** Average suppression slope plotted as a function of normalized suppression strength of pyramidal cell responses during stimulation of SOM+ neurons. The last point is near zero because the response is almost completely suppressed. **(c)** The zero-crossing point (threshold) plotted as a function of normalized suppression strength. These points indicate the 'floor effect' and degree of response subtraction evoked by SOM+ neurons. **(d)** Neuron distribution as a function of normalized suppression strength following SOM+ ChR2-activation. The suppression evoked ChR2-mediated spikes **(Supplementary Fig. 7)** spans a wide range but is biased toward lower values. **(e)** Distribution of the time-to-peak of pyramidal neuron responses during the control condition as a function of the suppression strength. The pyramidal cells that are strongly suppressed and display

a strongly divisive effect (low suppression slopes) are also the neurons that respond with a larger time-to-peak (***p<0.001, two-tailed t-test, between the two first (n=169) and two last bars (n=12)). The average time-to-peak of the most suppressed pyramidal neurons (0.33 sec, last 2 bars) is similar to the average time-to-peak of SOM+ neurons for sparse noise stimuli (0.34 sec, **Fig. 3d**), indicating that pyramidal cells that are co-active with SOM+ neurons are divisively suppressed. **(f)** Same as **e** for PV+ neurons. The relationship in **e** above does not hold for PV+ neurons, that are usually co-active with pyramidal neurons (N.S., Not Significant, p=0.54, two-tailed t-test, between the two first (n=38) and the two last bars (n=12)). Error bars indicate standard error of the mean.

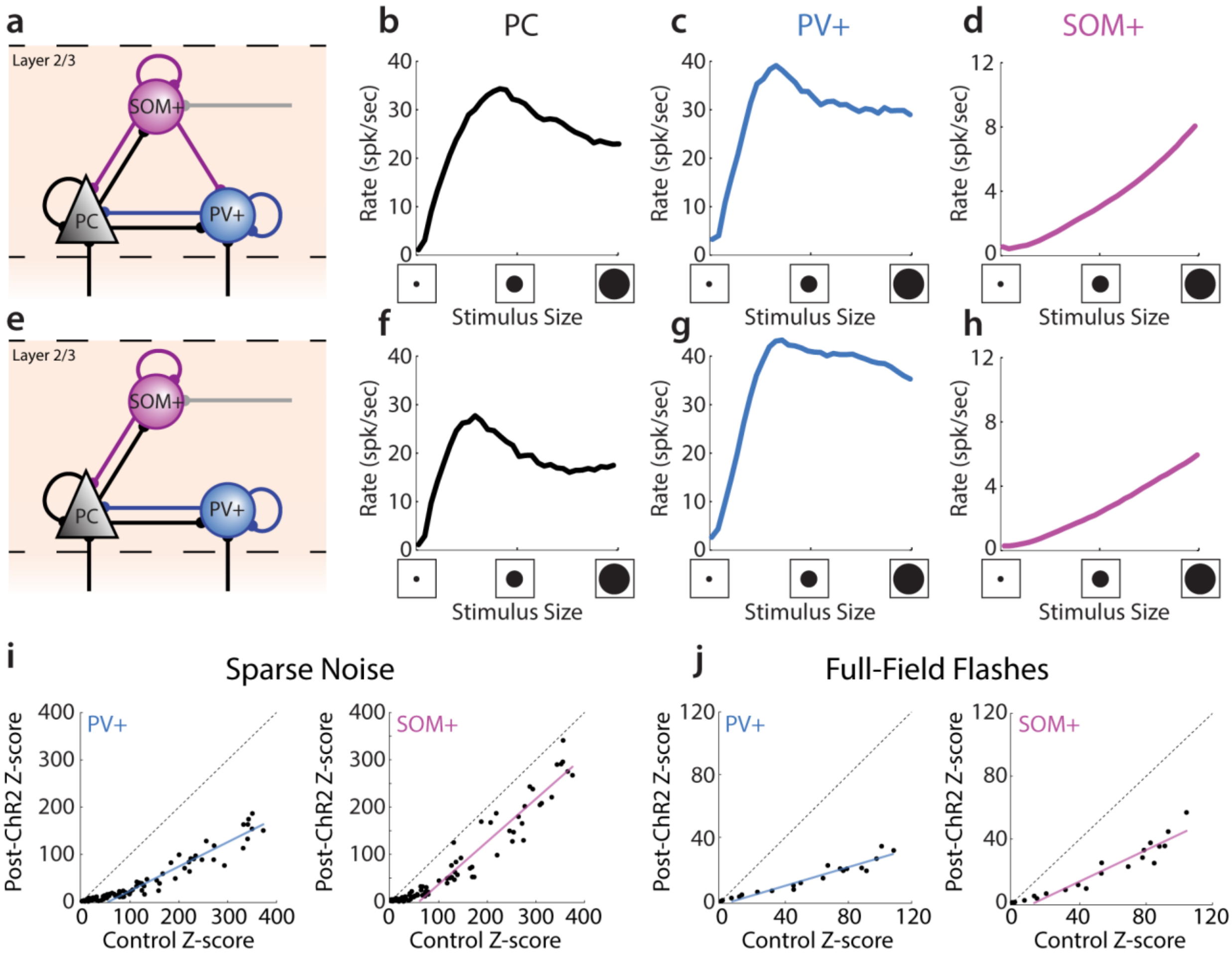

Supplementary Figure 12

Supplementary Figure 12. Effect of SOM+ to PV+ connections in producing surround suppression and stimulus-specific inhibition in the network model. **(a)** Schematic of the network connectivity where projections from SOM+ onto PV+ neurons were included. **(b-d)** Size tuning curves measured for pyramidal cells **(b)**, PV+ neurons **(c)** and SOM+ neurons **(d)**. Size tuning curves were assessed with increasing stimulated areas with fixed intensities. **(e-h)** Same as **a-d** but without projections from SOM+ to PV+ neurons. **(i)** The effect of PV+ (left, slope=0.52) and SOM+ (right, slope=0.91) activation on pyramidal cells for the network shown in **e** and during presentation of small stimuli (sparse noise). Each point of the corresponding receptive fields is compared between the control condition and when inhibitory neurons are activated. PV+ neurons are stimulated with a pulse conductance of 20nS and SOM+ neurons with 40nS. **(j)** Same as **i** but during full-field flashes (Slope=0.3 for PV+ and Slope=0.5 for SOM+). Connections from SOM+ to PV+ neurons were not required for the results of text **Fig. 7**, as shown in panels **i** and **j**.

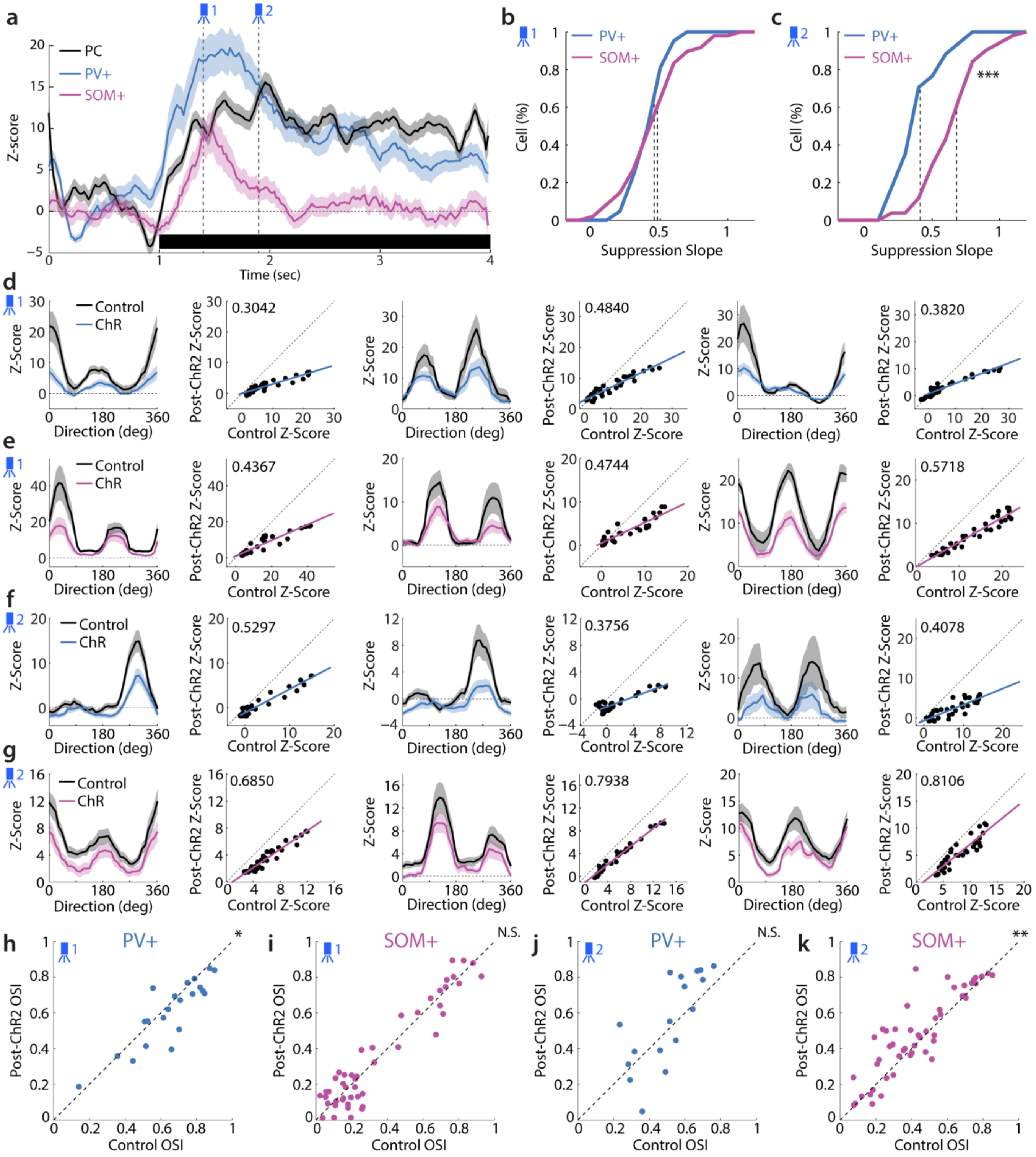

Supplementary Figure 13

Supplementary Figure 13. Effect of ChR2-mediated activation of PV+ or SOM+ neurons on target cell responses evoked by drifting gratings. The response temporal profile of PV+ and SOM+ neurons was measured by targeted cell-attached recordings during presentation of drifting gratings at 36 different directions. Similar experiments were performed in awake head-fixed mice to measure the response properties of putative pyramidal cells. **(a)** Population z-score response

averaged over all neurons and orientations for PV+ (blue, n=7), SOM+ (pink, n=9) and pyramidal cells (black, n=38). The black bar indicates the duration of the visual stimulation (3sec). PV+ and pyramidal cells displayed sustained firing, whereas SOM+ neurons responded to the stimulus onset only. We performed two sets of experiments where we respectively delivered the blue light pulse at 0.4 sec or 0.9 sec (vertical dashed lines) and compared the effect of PV+ and SOM+ neuron stimulation at these two time points. **(b-c)** Cumulative distributions of the linear fit slope for all cells that displayed a significant suppression ($p<0.01$, Wilcoxon signed-rank test) in response to PV+ (blue) or SOM+ (pink) activation at 0.4 sec (**b**, n=21 for PV+ and n=48 for SOM+, $p=0.64$, unpaired t-test) or at 0.9 sec after stimulus onset (**c**, n=17 for PV+ and n=51 for SOM+, $p<0.001$, unpaired t-test). Average values are indicated by dashed lines. **(d-e)** Comparison of individual cells' direction tuning curves between the control condition and when PV+ **(d**, r=0.92, r=0.95, r=0.96, $p<0.001$, paired t-test**)** or SOM+ **(e**, r=0.93, r=0.92, r=0.97, $p<0.001$, paired t-test**)** interneurons were activated with a single pulse at 0.4 sec after stimulus onset. In each panel, the slope of the fitted line is indicated. **(f-g)** Same as **d-e** but with the single pulse stimulation at 0.9 sec after stimulus onset. **(f)** r=096, r=0.92, r=0.85, $p<0.001$, paired t-test. **(g)** r=0.96, r=0.99, r=0.91, $p<0.001$, paired t-test. **(h-k)** Comparison of the orientation selectivity index (OSI) measured for individual cells between the control condition and when PV+ **(h,j)** or SOM+ **(i,k)** neurons were activated at 0.4 sec **(h,i)** or 0.9 sec **(j,k)** after onset of the drifting grating stimulus. * $p<0.05$; ** $p<0.01$; N.S. not significant, Wilcoxon signed-rank test.

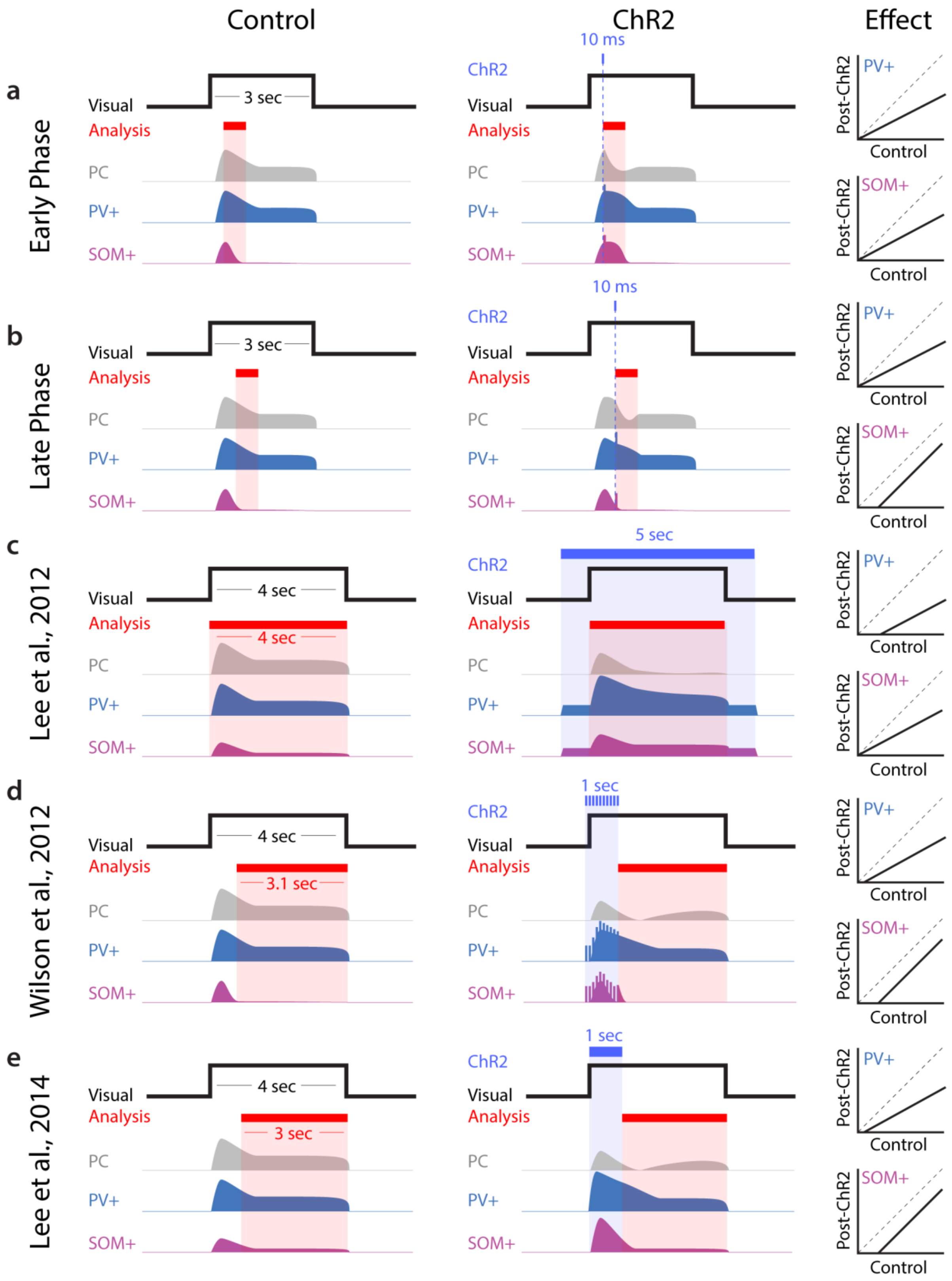

Supplementary Figure 14

Supplementary Figure 14. Comparison with previous published results. **(a-b)** Schematic of the experimental protocols used in **Supplementary Figure 13** for ChR2 stimulation coincident with

drifting gratings, during early phase **(a)** and late phase **(b)** of responses. These protocols are compared to previous work performed by Lee et al., 2012[8] **(c)**, Wilson et al., 2012[9] **(d)** Lee et al., 2014[10] **(e)**. For each experiment, the left panel illustrates the protocol in the control condition and the middle panel illustrates the protocol when PV+ or SOM+ neurons are activated with ChR2. The right panel describes the resulting effect on pyramidal cell responses by comparing schematic responses post-ChR2 with control responses. For each experiment and condition, the visual stimulation period is depicted by a black line, the segment of the recordings used for analysis is delimited by a red bar and the typical response modes are shown schematically for pyramidal (gray), PV+ (blue) and SOM+ (pink) cells. In Wilson et al. 2012[14], the initial part of the recordings was included in the calculation of orientation tuning curves only for cell-attached experiments and not for calcium imaging. In the middle panels, ChR2 stimulation is indicated as pulses (Wilson et al. 2012[14]) or continuous illumination (Lee et al., 2012[8] and Lee et al., 2014[10]).

SUPPLEMENTARY REFERENCES

1. Vogelstein, J. T. *et al.* Fast nonnegative deconvolution for spike train inference from population calcium imaging. *J. Neurophysiol.* **104,** 3691–3704 (2010).

2. Bonin, V., Histed, M. H., Yurgenson, S. & Reid, R. C. Local diversity and fine-scale organization of receptive fields in mouse visual cortex. *J. Neurosci.* **31,** 18506–21 (2011).

3. Hofer, S. B. *et al.* Differential connectivity and response dynamics of excitatory and inhibitory neurons in visual cortex. *Nat. Neurosci.* **14,** 1045–1052 (2011).

4. Smith, S. L. & Häusser, M. Parallel processing of visual space by neighboring neurons in mouse visual cortex. *Nat. Neurosci.* **13,** 1144–1149 (2010).

5. Liu, B. *et al.* Visual receptive field structure of cortical inhibitory neurons revealed by two-photon imaging guided recording. *J. Neurosci.* **29,** 10520–10532 (2009).

6. Huberman, A. D. & Niell, C. M. What can mice tell us about how vision works? *Trends Neurosci.* **34,** 464–473 (2011).

7. Hübener, M. Mouse visual cortex. *Curr. Opin. Neurobiol.* **13,** 413–420 (2003).

8. Lee, S.-H. *et al.* Activation of specific interneurons improves V1 feature selectivity and visual perception. *Nature* **488,** 379–383 (2012).

9. Wilson, N. R., Runyan, C. a, Wang, F. L. & Sur, M. Division and subtraction by distinct cortical inhibitory networks in vivo. *Nature* **488,** 1–6 (2012).

10. Lee, S.-H., Kwan, A. C. & Dan, Y. Interneuron subtypes and orientation tuning. *Nature* **508,** E1–2 (2014).
